# Supplementary material for: Large-scale whole-exome sequencing association studies identify rare functional variants influencing serum urate levels
Source: Nat Commun. 2018 Oct 12;9:4228. doi: 10.1038/s41467-018-06620-4 (PMC6185909; doi:10.1038/s41467-018-06620-4)
Supplement: Supplementary file 1 — Description of Additional Supplementary Files [file 41467_2018_6620_MOESM1_ESM.docx]

**File Name:** Supplementary Data 1
**Description:** Study population characteristics.

**File Name:** Supplementary Data 2
**Description:** Post-hoc power analysis results.

**File Name:** Supplementary Data 3
**Description:** Variants with p-value < 1e-07 in the primary single variant meta-analysis of eGFR. The results in the primary single variant meta-analysis of UACR are also shown.

**File Name:** Supplementary Data 4
**Description:** Genes with SKAT-O p-value < 1e-6 in the primary gene-based analysis of eGFR.

**File Name:** Supplementary Data 5 **Description:** Variants with p-value < 1e-07 in the primary single variant meta-analysis of UACR. The results of the variants from the primary single variant meta-analysis of eGFR are also shown.

**File Name:** Supplementary Data 6 **Description:** Genes with SKAT-O p-value < 1e-6 in the primary gene-based meta-analysis of UACR.

**File Name:** Supplementary Data 7
**Description:** Significant variants in the interrogation of the primary single variant meta-analysis results of eGFR.

**File Name:** Supplementary Data 8
**Description:** Significant variants in the interrogation of the primary single variant meta-analysis results of UACR.

**File Name:** Supplementary Data 9
**Description:** Variants with p-value < 1e-07 in the primary single variant meta-analysis of serum urate. The results of the variant in the primary single variant meta-analysis of gout are also shown.

**File Name:** Supplementary Data 10
**Description:** Conditional analysis of exome-wide significant variants in SLC2A9 and SLC22A12 in the ARIC study.

**File Name:** Supplementary Data 11
**Description:** Association of rs150255373 and rs147647315 with systolic blood pressure.

**File Name:** Supplementary Data 12
**Description:** Genes with SKAT-O p-value < 1e-6 in the primary gene-based meta-analysis of serum urate.

**File Name:** Supplementary Data 13
**Description:** Results in the primary meta-analysis of serum urate for low frequency or rare coding variants in SLC2A9 and SLC22A12 associated with serum urate reported in the literature.

**File Name:** Supplementary Data 14
**Description:** Significant genes in the interrogation of the serum urate primary gene-based metaanalysis results.

**Name:** Supplementary Data 15
**Description:** Significant variants in the interrogation of the serum urate primary single variant meta-analysis results.

**File Name:** Supplementary Data 16
**Description:** Genetic variants at SLC22A12 that were selected for function study.

**File Name:** Supplementary Data 17
**Description:** Study design, acknowledgements, and assay methods of serum creatinine, UACR, serum urate, and gout.

**File Name:** Supplementary Data 18
**Description:** Study specific exome sequencing summary

**File Name:** Supplementary Data 19
**Description:** Population characteristics and genotype imputation information of rs150255373 in CoLaus and SHIP-trend.

**File Name:** Supplementary Data 20
**Description:** Disease traits included in GWAS catalog lookup and phenotypes used in identifying kidney function or urate trait-associated genes in genetically manipulated mouse model.

**File Name:** Supplementary Data 21
**Description:** GWAS loci of serum urate and kidney function used in the interrogation of the primary meta-analysis results.

**File Name:** Supplementary Data 22
**Description:** Genes used in the interrogation of the primary meta-analysis results of kidney function and serum urate.

**File Name:** Supplementary Data 23
**Description:** Significance threshold for interrogation of the primary meta-analysis results for GWAS loci, genes linked to serum urate or kidney function dysregulation in Mendelian disease or mouse model studies.

**File Name:** Supplementary Data 24
**Description:** Putative damaging variants in SLC2A9 selected for mapping into SLC2A9 structure.
