# Supplementary material for: Large-scale whole-exome sequencing association studies identify rare functional variants influencing serum urate levels
Source: Nat Commun. 2018 Oct 12;9:4228. doi: 10.1038/s41467-018-06620-4 (PMC6185909; doi:10.1038/s41467-018-06620-4)
Supplement: Supplementary file 2 — Supplementary Information [file 41467_2018_6620_MOESM2_ESM.pdf]

Large-Scale Whole-Exome Sequencing Association Studies Identify Rare Functional Variants Influencing Serum Urate Levels

Tin A, *et al*

Supplementary Information

Supplementary Methods

Secondary meta-analyses. To assess potential heterogeneity between race groups, we conducted race-specific single variant and SKAT-O meta-analyses using the same variant filtering criteria and methods as the primary meta-analysis (**Supplementary Figure 10**). To evaluate whether a score integrating additional annotation sources might improve association signals, we conducted race-specific and race-combined SKAT-O meta-analyses that included all putative damaging variants regardless of MAF weighted by their Combined Annotation–Dependent Depletion (CADD) scores.[1] For genes that were significantly associated with serum urate in the primary meta-analysis, we conducted a burden test for gout using uniform weight to evaluate the association per copy of variant.

Follow-up analysis of urate-associated rare variants and blood pressure

Given the long standing controversy around the potential influence of serum urate on blood pressure,[2-4] the association between the rare variants with large effect on serum urate and systolic blood pressure (SBP) was evaluated in the ARIC study, overall and stratified by sex. Any significant variants ($p<0.05$) were evaluated for replication in an exome-wide association study of blood pressure,[5] and the CoLaus and SHIP-Trend studies (**Supplementary Data 19**).[5-7] The methods of this SBP association analysis were the same as in Yu et al.[5] Briefly, if a participant was treated for hypertension, 15 mm Hg was added to SBP levels. Participants with SBP <60 mm Hg without hypertension treatment were excluded. SBP levels were further winsorized at the 99.9th percentile. We performed inverse variance weighted fixed effect meta-analysis to combine the results from all replication studies as well as from ARIC and the replication studies. The replication significance was set at p-value <0.05.

Interrogation of primary meta-analysis results for urate-associated coding variants in *SLC2A9* and *SLC22A12*. We conducted a search for reported coding variants associated with serum urate in *SLC2A9* and *SLC22A12* using the keywords: “human”, “mutation”, and “urate”, combined with “*SLC2A9*“ or “*SLC22A12*“. Then we excluded publications that were non-English or reviews, reported only known variants, functional or lab method studies, or AKI only studies. Altogether 21 publications were identified.[8-28] We then interrogated the primary single variant meta-analysis results of serum urate for the coding variants found in the 21 publications.

Interrogation of primary meta-analysis results for candidate variants and genes from GWAS, Mendelian diseases, and mouse models. We investigated whether genome-wide significant loci identified from GWAS might harbor low frequency or rare variants associated with our outcomes. Index variants of known loci for serum urate, eGFR and UACR were identified using the NHGRI-EBI Catalog of Published Genome-wide Association Studies[29] based on the following criteria: i) disease traits that matched the selected kidney function or serum urate related traits in **Supplementary Data 20**; ii) index variants with p-value $<5x10^{-8}$ with replication; iii) in studies of adult populations. The list of GWAS index SNPs identified are reported in **Supplementary Data 21** for eGFR, UACR and serum urate. In addition, genes that have been linked to Mendelian forms of kidney dysfunction or serum urate dysregulation were evaluated for associated low frequency or rare variants in our population-based studies. These genes were identified based on a comprehensive gene panel for diagnosis of genetic nephropathies (**Supplementary Data 22**).[30] Genes linked to Mendelian forms of serum urate dysregulation were identified using the Online Mendelian Inheritance in Man (OMIM)[31] database by searching for phenotypes with known molecular basis linked to hyperuricemia or hypouricemia (**Supplementary Data 22)**. Finally, genes linked to kidney function or serum urate dysregulation in

genetically manipulated mice were retrieved from the Mouse Genome Informatics (MGI) database using previously described methods.[32; 33] Briefly, phenotype ontology terms related to abnormal kidney function or serum urate in genetically manipulated mice were selected (**Supplementary Data 20)**, followed by linking to the human ortholog through the Human-Mouse:Disease Connection (HMDC) database.[34] The resulting genes for abnormal kidney morphology, for abnormal kidney physiology, and for abnormal urate levels are also listed in **Supplementary Data 22**. The significance thresholds for the interrogation of the primary meta-analysis results for variants mapping to these genes were determined by the Bonferroni method as reported in **Supplementary Data 23**.

Selection of variants for experimental studies. To prioritize variants detected through WES in *SLC22A12* for experimental studies of the encoded urate transporter URAT1, we established a protocol to identify the variants that were most likely to have functional or regulatory effects based on the long isoform and the corresponding mRNA amino acid sequence (NP_653186; **Supplementary Figure 2)**. Using an in-depth literature search, we created maps of the predicted transmembrane domains and other domains or elements that may have functional or regulatory consequences. The amino acid sequence was subjected to a bioinformatic analysis of phosphorylation sites using GPS 3.0,[35] a program that evaluates the probability that any given S, T, or Y is phosphorylated based on known kinase binding motifs. Finally, we produced two protein sequence alignments: one with other human protein sequences of transporters of the same family; and a second with the same protein in a representative sampling of other vertebrates. We compared sequence conservation and looked for discordant conservation in the two alignments as a potential indicator of urate specific function or regulation. After our literature based "maps" were constructed, we then located the newly identified variants and looked for

those in areas of predicted functional or regulatory significance. **(Supplementary Data 16).**

Molecular Biology. Primers used for SLC22A12 mutagenesis: K536T FW 5’ GAA CCA GGC AGT AAC GAA GGC AAC ACA TG 3’, RV 5’ CAT GTG TTG CCT TCG TTA CTG CCT GGT TC 3’; R405C FW 5’ GAG CCA CCT GGG CTG CCG CCC CAC GCT G 3’, RV 5’ CAG CGT GGG GCG GCA GCC CAG GTG GCT C 3’; T467M FW 5’ GTG CTC AGG ATG ATG GCA GTG GGC TTG 3’, RV 5’ CAA GCC CAC TGC CAT CAT CCT GAG CAC 3’; R325W FW 5’ CTT TCA GCC ATG TGG GAG GAG CTG AG 3’, RV 5’ CTC AGC TCC TCC CAC ATG GCT GAA AG 3’.

Supplementary Note 1

Variants in known kidney-function associated genes are associated with eGFR and UACR

Single variant analysis of eGFR confirmed known associations of common variants in *GATM,* its neighboring gene *SPATA5L1* as well as in *CPS1* as exome-wide significant (**Supplementary Data 3**).[36] These eGFR-associated variants were not associated with UACR (p>0.05). The SKAT-O meta-analysis of eGFR with MAF <5% confirmed the known association of low frequency and rare variants in *SLC47A1* ($p=1.1x10^{-6}$, **Supplementary Data 4**).[37]

Single variant meta-analysis of UACR confirmed the known association of rs334, the sickle cell trait variant at *HBB* among populations of African ancestry, as exome-wide significant (**Supplementary Data 5 and 6**).[38] The A allele, which was associated with higher UACR, was also nominally associated with lower eGFR ($p=6.1x10^{-4}$).

Overall, WES association analyses in 18,800 population-based individuals did not identify any low frequency or rare variants associated with eGFR or UACR that mapped outside of known GWAS loci.

Interrogation of eGFR and UACR primary meta-analysis results for candidate variants or genes implicated in GWAS of eGFR, Mendelian form of kidney disease, and abnormal kidney morphology and physiology based on genetically manipulated mouse models.

We interrogated the primary meta-analysis results of eGFR and UACR for variants in known GWAS loci, genes linked to Mendelian form of kidney disease, and genes linked to abnormal kidney function from genetically manipulated mouse models listed in **Supplementary Data 20** based on significance thresholds reported in **Supplementary Data 23**. Interrogation of the eGFR primary single variant meta-analysis results identified significant eGFR-associated common variants in the following genes: *CPS1*, *GATM*, and *SLC28A2* among the 56 eGFR GWAS loci ($p < 6.4 \times 10^{-6}$); the neighboring *ALMS1* and *NAT8* at $p < 1.2 \times 10^{-5}$ among the 301 genes linked to Mendelian form of kidney disease; and *ALSM1* among the 366 genes linked to abnormal murine kidney physiology (**Supplementary Data 7**). Interrogation of the UACR primary single variant meta-analysis results confirmed the association of *APOL1* risk variant rs73885319 (G1) with increased UACR (**Supplementary Data 8**). Interrogation of primary SKAT-O results of eGFR and UACR did not identify any significant associations.

Follow-up analysis on the association between urate-associated rare variants at *SLC22A12* and blood pressure

Given the large effect of rs150255373 and rs147647315 on serum urate levels and the long-standing debate around the relationship of serum urate levels and blood pressure,

we conducted association analyses of these variants and SBP in the ARIC study. Consistent with the reported relationship of hyperuricemia and hypertension, the urate-lowering T allele of rs150255373, found exclusively in EA, was significantly associated with lower SBP in ARIC EA overall and in males (overall: n=7,391, beta=-8.2 mm Hg, p=0.03; males: n=3,421, beta=-18.1 mm Hg, p=0.004). The A allele of rs147647315, found largely in AA, was not significantly associated with SBP in ARIC AA participants (p>0.7 in AA overall and sex-stratified analysis, **Supplementary Data 11**). In replication studies, the association between rs150255373 and SBP was in the same direction but not significant (overall: n=16,533, beta=-5.0 mm Hg, p=0.16; males: n=6,520, beta=-2.2 mm Hg, p=0.69, **Supplementary Data 11**).

Interrogation of serum urate primary meta-analysis results for candidate variants or genes implicated in GWAS of serum urate, abnormal serum urate levels based on Mendelian inheritance or genetically manipulated mouse models.

In the interrogation of the single variant primary meta-analysis results of serum urate for 31 urate-associated GWAS loci, 34 genes linked to Mendelian forms of abnormal urate levels, and 36 genes linked to abnormal urate levels from genetically manipulated mouse models, we confirmed the association of common variants in 8 genes and no additional low frequency or rare variants were identified (**Supplementary Data 15**). In the interrogation of the SKAT-O primary meta-analysis results of serum urate in the above 3 sets of genes or regions, in addition to confirming the association of *SLC22A12* and *SLC2A9*, *HPRT1* on chromosome X was identified as associated with serum urate levels among 34 genes linked to Mendelian form of abnormal urate levels (SKAT-O $p=1.4x10^{-3}$, **Supplementary Data 14**). This is the first instance that a variant in a gonosomal gene was identified as associated with serum urate in population-based association studies.

Supplementary Note 2 Analysis Plans

Exome Sequencing Analysis Plan for Serum Urate and Gout
February 2015

Please contact Adrienne Tin (atin@jhsph.edu) or Anna Kottgen (anna.koettgen@uniklinik-freiburg.de) if you have any questions regarding this analysis plan.

Purpose of this analysis plan:

- This analysis plan is for generating score statistics into an R object using data from one cohort. The meta-analysis team will use the score statistics in the R object to perform meta-analyses for single SNP test, gene-based burden and SKAT tests. The functional and allele frequency criteria for variants in the gene-based tests will be decided at the meta-analysis stage.

General guideline for all analyses:

- Perform all analyses stratified by self-reported race, European American (EA) vs. African American (AA).
- Restrict analyses to individuals with available information on urate, sex, age, and BMI for the urate analyses, and gout, age, sex for the gout analyses (include study center or cohort status if applicable)
- Please account for individual relatedness in the appropriate way if necessary.
- Individual studies should adjust for measures of population stratification through the use of principal components if available and necessary.
- Please do not exclude individuals with gout (treated or not) from the analyses of serum urate.
- Serum urate measures should be in mg/dL. To convert umol/L to mg/dL, please divide by 59.48. Your mean serum urate should be approximately 5-6.5 mg/dl. Please do not impute missing values of urate.
- Include all variants in your data, even if they are monomorphic.
- Assume additive genetic model.

Phenotype definitions:

Please use the following definitions to define gout in cohorts that have this information available:

a. Self-report of gout at a study exam or a questionnaire. If not available, please use:
b. Gout defined based on ICD-codings (ICD-9 code 274.0, 274.1, 274.8, or 274.9; ICD-10 M10.0, M.10.3, M.10.4, M10.9) from hospital discharge records or death certificates. If not available, please use:
c. Intake of previous or current gout-specific medication: probenecid, benzbromarone, sulfinpyrazone, pegloticase, rasburicase, febuxostat or allopurinol
d. If you are using another definition, please let us know what this definition is in the accompanying excel spreadsheet.

Please provide information on proportion with gout and gout case counts in the excel spreadsheet even if the number of gout cases is too small for analysis (<50 cases).

- Use the following definitions for diabetes and hypertension for **Table 1** information:

- **Diabetes**:
  - **Preferred definition:** fasting plasma glucose ≥ 126 mg/dl (7.0 mmol/L) OR treatment for diabetes
  - **If fasting glucose is not available:** casual glucose (non-fasting) ≥ 200 mg/dl (11.0 mmol/L) OR treatment for diabetes
  - **If glucose is not available:** self-reported diabetes status
  - **If an alternative definition was used:** please specify this in your methods write-up
- **Hypertension**:
  - **Preferred definition:** systolic blood pressure ≥ 140 mm Hg OR diastolic blood pressure ≥ 90 mm Hg OR treatment for hypertension
  - **If measured blood pressure is not available:** self-reported hypertension.
  - **If an alternative definition was used:** please specify in your methods write-up

Analysis steps

Step 1: Download seqMeta package and snp information file.

A) The **seqMeta** package (version 1.6.5) can be downloaded from CRAN.
B) Version 13 of the SNP information file can be downloaded from the 'Annotation' folder in Google Drive: (https://drive.google.com/drive/folders/0BzYDtCo_doHJT0JVeXYyRXh2NmM)

Step 2: Perform inverse normal transformation of the adjusted residuals of serum urate as outcome. The covariates should include age, sex, BMI, appropriate PCs, and study centers if applicable.

```
    # Example R code for inverse normal transformation of the
adjusted residuals of serum urate
        library("GenABEL")
        urate_resid = resid(lm(urate~age+sex+BMI+pc1+pc2))
        urate_resid_invnorm = rntransform(urate_resid)
```

Step 3: Generate score statistics using the prepScores function (for chromosome X, use prepScoresX and code the genotypes in male as 0/2) in the seqMeta R Package with the inverse normal transformed residuals of serum urate as a continuous outcome. Some example R code are provided at the end of the analysis plan.

a. **Please conduct all analyses by chromosome because different chromosomes may have genes with the same name that should be aggregate separately.**
b. **prepScores** will generate the score statistics for all SNPs. Please upload the results as Rdata file.
   I. The outcome cannot contain missing value.
   II. Individual data for genotype and outcome need to be in the same order.
   III. Use the SNP information file downloaded in **Step 1** to define the aggregation of the SNPs.
   IV. The coding allele of the genotype needs to be the same as the alternate (ALT) allele in the snpinfo file.

   V. In the regression model, include adjustment for relatedness if necessary. An example of the regression model using the inverse normal transformed

residuals of urate as outcome in R syntax is provided in the example code below (see command 2).

c. Save the Rdata file using the following naming convention (one output for each chromosome in each ethnic group):

[YOURSTUDYNAME]_ES_URATE_INVNORM_[RACE]_chr[N]_[MMDDYYYY].Rdata where

RACE is either EA or AA;
N=1 to 22 and X for the chromosomes.
MMDDYYYY is the time stamp.

For example, the file for chromosome 1 in European Americans in the ARIC study is:
ARIC_ES_URATE_INVNORM_EA_chr1_02132015.Rdata

Step 4: Generate score statistics using the prepScores function (for chromosome X, use prepScoresX and code the genotypes in male as 0/2) in the seqMeta R Package with gout as a binary outcome. Some R code examples are provided at the end of the analysis plan.

a. **prepScores** will generate the score statistics for all SNPs. Please upload the results as Rdata file.
   I. The outcome cannot contain missing value.
   II. Individual data for genotype and outcome need to be in the same order.
   III. Use the SNP information file downloaded in **Step 1** to define the aggregation of the SNPs.
   IV. In the regression model, include adjustment for relatedness if necessary. An example of the regression model in R syntax is provided in the example code below (see command 2).

b. Save the Rdata file using the following naming convention (one output for each chromosome in each ethnic group):

[YOURSTUDYNAME]_ES_GOUT_[RACE]_chr[N]_[MMDDYYYY].Rdata where

RACE is either EA or AA;
N=1 to 22 and X for the chromosomes.
MMDDYYYY is the time stamp.

For example, the file for chromosome 1 in European Americans in the ARIC study is:
ARIC_ES_GOUT_EA_chr1_02132015.Rdata

Step 5. Upload cohort results. Each outcome, e.g. residuals of serum urate, will have 23 files for each ethnic group (one for each chromosome). For file upload, please zip up the output for one outcome into 1 or 2 files.

Example R code for the cohort level analysis:

```
Command 1: Load CHARGE SNP information file
snpinfo = get(load("snpinfo_WES_v13_Analytic_ChrN_10202015.Rdata"))
```

Command 2A: Create a seqMeta object containing one cohort's contributions for unrelated individuals

```
# for residuals of serum urate
cohort_score_urate = prepScores(Z = genos_chrN, formula =
"urate_resid_invnorm ~ 1", data = pheno, SNPInfo = snpinfo,
snpNames="SNP", aggregateBy = "SKATgene")

# for gout
cohort_score_gout = prepScores(Z = genos_chrN, formula = "gout ~ age +
sex + pc + center", family=binomial(), data = pheno, SNPInfo = snpinfo,
snpNames="SNP", aggregateBy = "SKATgene")
```

Command 2B: Create a seqMeta object containing one cohort's contributions for related individuals

```
# for residuals of serum urate
cohort_score_urate = prepScores(Z = genos_chrN, formula =
"urate_resid_invnorm  ~ 1", fullkins = makekinship(fullped$fam,
fullped$id, fullped$fa, fullped$mo), data = pheno, SNPInfo = snpinfo,
snpNames="SNP", aggregateBy = "SKATgene")

# for gout
cohort_score_gout = prepScores(Z = genos_chrN, formula = "gout ~ age +
sex + pc + center", family=binomial(),fullkins =
makekinship(fullped$fam, fullped$id, fullped$fa, fullped$mo), data =
pheno, SNPInfo = snpinfo, snpNames="SNP", aggregateBy = "SKATgene")
```

Command 3: Save the result as Rdata file

```
save(cohort_score_urate,file =
"COHORTA_ES_URATE_INVNORM_EA_chrN_02132015.Rdata")

save(cohort_score_gout,file = "COHORTA_ES_GOUT_EA_chrN_02132015.Rdata")
```

After you have performed these analyses. Please fill in the attached tables and upload your results files within 4 weeks to the following Google Drive:

https://drive.google.com/drive/folders/0BzYDtCo_doHJM0dwZ3dqSEZxZkU
CHARGE – Urate exome sequencing

To access this Google Drive:

1) Sign up for a Google Account ID (username@gmail.com) and password at: http://accounts.google.com/SignUp

2) E-mailed your Google Account ID, your study name and the requested Google Drive (CHARGE – Urate exome sequencing) to chargeco@u.washington.edu and you will be added to the access list (please cc Anna Kottgen (anna.koettgen@uniklinik-freiburg.de). Once your ID has been added, you will receive an email from chargeco.

Renal Trait Analysis Plan using Genotypes from Exome Sequencing
February 2016

This analysis plan for exome sequence genotype is based on our prior trait creation so that we can remain consistent with our prior work. The traits of interest are eGFRcrea, eGFRcys, UACR, and CKD. (Details for CKD will be specified in a later time because the methods for controlling type 1 errors for gene-based test of rare variants for binary traits are still in development.)

Please contact Anna Kottgen (anna.koettgen@uniklinik-freiburg.de) and Adrienne Tin (atin1@jhu.edu) if you have any questions regarding trait creation or variable definitions.

General guideline for all analyses:

- Perform all analyses stratified by self-reported race (European American [EA] vs. African American [AA]).
- If different phenotypes were collected at different times in your study, please use the **covariates** from the respective time point of the main phenotype collection in the analyses. For example, if eGFRcrea was collected at visit 4 of the study, please use age at visit 4 for that analysis.
- If you have more than one visit with measurement of serum creatinine, use the visit with the largest sample size. This applies to all other traits where you may have more than one measure; we would like to maximize sample size.
- All genotypes are called from the + strand using hg19 as the reference and the reference and alternate alleles need to match the alleles in the CHARGE SNP information file
- Please include information on all SNPs in your sample, even if they are monomorphic
- The numbers in Table 1 should refer to the time point of serum creatinine measurement used to calculate eGFRcrea overall.
- Fill in Table 1 as you go along; please use the following definitions to define diabetes and hypertension for Table 1 information:
    - **Diabetes** should be defined as:
        - **Preferred definition:** fasting plasma glucose ≥ 126 mg/dl (7.0 mmol/L) OR treatment for diabetes
        - **If fasting glucose is not available:** casual glucose (non-fasting) ≥ 200 mg/dl (11.0 mmol/L) OR treatment for diabetes
        - **If glucose is not available:** self-reported diabetes status
        - **If an alternative definition was used:** please specify this in your methods write-up
    - **Hypertension** should be defined as:
        - **Preferred definition:** systolic blood pressure ≥ 140 mm Hg OR diastolic blood pressure ≥ 90 mm Hg OR treatment for hypertension
        - **If measured blood pressure is not available:** self-reported hypertension.
        - **If an alternative definition was used:** please specify in your methods write-up
    - **Define CKD as eGFRcrea<60**

Step by Step Analysis Plan:

<u>TRAIT CREATION (STEPS 1 TO 5).</u> These trait definitions are the same as those in the exome chip analysis plan.

<u>Step 1</u>: **Calibrate the serum creatinine.** The goal is to have a similar mean serum creatinine for each age group to the one in NHANES, a nationally representative US survey. Here are the SAS commands for the calibration. **START BY MAKING SURE YOUR CREATININE IS IN MG/DL units.** If your creatinine is in μmol/l, please divide by 88.4 to obtain mg/dl. Your mean creatinine should be approximately 0.7-1.1 mg/dl.

For European ancestry participants, use Step 1a.
For African ancestry participants, use Step 1b

<u>Step 1a</u>: SAS code for calibrating the serum creatinine in European ancestry participants

```
/* Notation:
age1= age, creatin1=creatinine,
sex = 1 for men and sex = 2 for women */

data analyze;
  set YOURDATA;
  if sex ne . and age1 >=20;  /* ne is SAS code for: not equal */
  if creatin1 >=0;
run;

data analyze;
  set analyze;
  if  20 <= age1 < 40  then agegrp=1;
  else if  40 <= age1 < 60  then agegrp=2;
  else if  60 <= age1 < 70  then agegrp=3;
  else if age1 >=70  then agegrp=4;
run;

proc sort; by sex agegrp; run;

proc means noprint;
  var creatin1; by sex agegrp;
  output out=meanc mean=meancrt;
run;

data analyze;
  set analyze;
  merge analyze meanc;
  by sex agegrp;
  /* generate calibrated means based on the NHANES agegroup-and sex-
specific means */
  if sex=1 and agegrp=1 then nhcreat1= creatin1 - meancrt + 0.9101763;
  else if sex=1 and agegrp=2 then nhcreat1= creatin1 - meancrt + 0.9347561;
  else if sex=1 and agegrp=3 then nhcreat1= creatin1 - meancrt + 0.9957202;
  else if sex=1 and agegrp=4 then nhcreat1= creatin1 - meancrt + 1.127975;
  else if sex=2 and agegrp=1 then nhcreat1= creatin1 - meancrt + 0.7016764;
  else if sex=2 and agegrp=2 then nhcreat1= creatin1 - meancrt + 0.7304324;
  else if sex=2 and agegrp=3 then nhcreat1= creatin1 - meancrt + 0.8134599;
  else if sex=2 and agegrp=4 then nhcreat1= creatin1 - meancrt + 0.8771303;
run;
```

```
data analyze;   /* Remove variables meancrt and agegrp, because they
are not needed in the following steps */
  set analyze;
  drop meancrt agegrp;
run;
```

Step 1b: SAS code for calibrating the serum creatinine in African ancestry participants

```
/* Notation:
age1= age, creatin1=creatinine,
sex = 1 for men and sex = 2 for women */

data outdat;
     set indat;
if age1 < 20  then  delete;
if creatin1 <0   then delete;
/* calculate gfr */;

if      20 <=  age1 < 40   then  agegrp=1;
else if   40 <=  age1 < 60   then  agegrp=2;
else if   60 <=  age1 < 70   then  agegrp=3;
else if         age1>= 70  then  agegrp=4;
run;

proc  sort; by sex agegrp;
proc  means noprint;
var  CREATIN1 ; by sex  agegrp;
output  out=meanc  mean=meancrt;
run;
proc print  data=meanc;

data  all; merge outdat meanc; by sex agegrp;
run;
data all;
     set all;

   if  sex=1  and agegrp=1  then  nhcreat1= CREATIN1  -meancrt + 1.00501;
else if  sex=1  and agegrp=2  then  nhcreat1= CREATIN1  -meancrt + 1.026986;
else if  sex=1  and agegrp=3  then  nhcreat1= CREATIN1  -meancrt + 1.132971;
else if  sex=1  and agegrp=4  then  nhcreat1= CREATIN1  -meancrt + 1.200909;
else if  sex=2  and agegrp=1  then  nhcreat1= CREATIN1  -meancrt + 0.7514;
else if  sex=2  and agegrp=2  then  nhcreat1= CREATIN1  -meancrt + 0.774523;
else if  sex=2  and agegrp=3  then  nhcreat1= CREATIN1  -meancrt + 0.905134;
else if  sex=2  and agegrp=4  then  nhcreat1= CREATIN1  -meancrt + 0.968041;
run;
```

Step 2a: Calculating eGFRcrea from the MDRD equation in European Ancestry participants. Be sure to use the calibrated serum creatinine measurements from Step **1a**.

$eGFRcrea = 186.3*(nhcreat1)^{-1.154} * age^{-0.203} * (0.742 \text{ if female})$.

Next, set all eGFRcrea values>200 to 200 and those <15 to 15.

```
/*SAS-Code:*/
data analyze;
  set analyze;
  if sex=1 then eGFRcrea1=186.3*(nhcreat1** (-1.154))*(age1** (-
0.203));
  else if sex=2 then eGFRcrea1=186.3*(nhcreat1**(-1.154))*(age1**(-
0.203))*0.742;
  if eGFRcrea1>200 then eGFRcrea1=200;
  if eGFRcrea1<15 then eGFRcrea1=15;
run;
```

<u>Step 2b</u>: Calculating eGFRcrea from the MDRD equation in African ancestry participants. Be sure to use the calibrated serum creatinine measurements from Step **1b**.

$$eGFRcrea = 186.3*(nhcreat1)^{-1.154} * age^{-0.203} * (0.742 \text{ if female})*(1.21 \text{ if African ancestry}).$$

Next, set all eGFRcrea values>200 to 200 and those <15 to 15.

```
/*SAS-Code:*/
data analyze;
  set analyze;
  if sex=1 then eGFRcrea1=186.3*(nhcreat1** (-1.154))*(age1** (-
0.203))*1.21;
  else if sex=2 then eGFRcrea1=186.3*(nhcreat1**(-1.154))*(age1**(-
0.203))*0.742*1.21;
  if eGFRcrea1>200 then eGFRcrea1=200;
  if eGFRcrea1<15 then eGFRcrea1=15;
run;
```

<u>Step 3</u>: define CKD as eGFRcrea<60. Please fill in eGFRcrea and CKD information into **Table 1**, along with information about age and sex.

<u>Step 4</u>: Calculate eGFRcys using cystatin C

a. Make sure your cystatin C is in mg/L
b. Create eGFRcys using the following equation

$egfrcys = 76.7*(\text{serum cystatin C})^{-1.19}$
if egfrcys >200 then egfrcys=200;
if egfrcys <15 then egfrcys=15;

```
/**SAS code**/
egfrcys = 76.7 * serumcys**(-1.19);
if egfrcys > 200 then egfrcys = 200;
else if egfrcys < 15 then egfrcys =15;
```

<u>General guideline for analyses of urinary albumin and urinary creatinine</u>

- Dealing with urine albumin levels below the assay's detection threshold: values that are below the detection limit of your particular urinary albumin assay should <u>not</u> be missing or zero.

- If you have urine albumin levels below the detection threshold, please note how often you observe this in your overall sample (proportion of total population with the lowest detection limit value) and the detection threshold value of the assay in Table 1.
- Please set the values below the detection limit to the lower limit of your urinary albumin assay; this value should not be ZERO. In our experience, the majority of studies use a urinary albumin assay with a lower limit of detection of 3 mg/L. If this applies to your study, then please set all values below this value of 3 mg/L to 3 mg/L. Your study may have used an assay with a different level of detection. If this is the case, then assign those with urinary albumin below the assay's detection threshold [<assay] to this level. Divide this new value by the urinary creatinine value to obtain an ACR value for each of the participants in your dataset.

Step 5: Create UACR

a. Define UACR as [urinary albumin(mg/L) / urinary creatinine(mg/dl)]*100; Important: your urinary albumin and urinary creatinine values may have different units, so adapt the formula as needed to obtain UACR measures in mg/g.
b. If your ACR is in the unit "mg/mmol", convert to mg/g by using the following information: 1g creatinine = 8.84mmol creatinine (or 1mg/dL creatinine = 88.4 μmol/L creatinine).

   For example, ACR of 3.4mg albumin / mmol creatinine is equivalent to 8.84x3.4mg = 30.056 mg albumin / g creatinine.

c. In general population studies, the median of ACR is usually around 1.5 mg/g.
d. Enter the median and 25% and 75% percentile values of your distribution into Table 1.

COHORT ANALYSES (STEPS 6 to 10)

Step 6: Perform two transformations of the traits: log transformation (Step 6a) and inverse normal transformation of the residuals from the log transformation (Step 6b).

Step 6a: Perform log transformation of eGFRcrea, eGFRcys, and UACR that you created in steps 1, 2, 4, and 5.

```
# Example R code for log transformation of eGFRcrea
eGFRcrea_log = log(eGFRcrea)
```

Step 6b: Perform inverse normal transformation of the residuals from the log transformation of the trait after regressing it against the covariates

```
# Example R code for inverse normal transformation of
log(eGFRcrea) residuals
library("GenABEL")
eGFRcrea_log = log(eGFRcrea)
egfr_log_resid = resid(lm(eGFRcrea_log~age+sex+pc1+pc2))
egfr_log_resid_invnorm = rntransform(egfr_log_resid)
```

Step 7: Perform cohort analyses of the log transformation of the traits (eGFRcrea, eGFRcys, and UACR) and inverse normal transformation of the residuals from the log transformation of the traits.

Please use ALL SNPs, including monomorphic ones, in the analysis. The SNP selection criteria for burden test and SKAT test, including MAF threshold and function, will be decided at the meta-analysis stage.

a. **Please conduct all analyses by chromosome because different chromosomes may have genes with the same name that should be aggregate separately.**

b. For the log transformation of the trait from **step 6a**, please include covariates (age, sex, study center, and PCs).
c. For the inverse normal transformation of the residuals from **step 6b**, please do not include covariates.
d. Perform analyses using the prepScores() function for chromosomes 1 to 22 and the prepScoresX() function for chromosome X in the seqMeta package (version 1.6.5 , cran.r-project.org/web/packages/seqMeta/), aggregate by SKATgene column in the SNP annotation file
e. For either prepScores or prepScoresX, the order of the individuals in the genotype and the phenotype files need to be identical.
f. The coding allele of the genotype needs to be the same as the alternate (ALT) allele in the snpinfo file.
g. Be sure to use the snpinfo files that include all variants discovered in your cohort. Version 13 of the snpinfo files can be downloaded from the 'Annotation' Google Drive folder (https://drive.google.com/drive/folders/0BzYDtCo_doHJT0JVeXYyRXh2NmM)
h. Studies with family data should use the kins argument to provide the kinship matrix for prepScores or prepScoresX
i. prepScoreX requires an additional parameter "male" for calculating allele frequency. The genotype for male at chromosome X should be coded as 0 or 2 to account for X-inactivation.
j. Perform all analyses stratified by ethnicity/race
k. Phenotype data cannot contain null values
l. Phenotype and genotype data need to be in the same order
m. Column names in the genotype data need to be the same as the SNP names in the SNP annotation data
n. Use the alternate allele as the coding allele and assume an additive genetic model
o. Save the Rdata file using the following naming convention:

[YOURSTUDYNAME]_ES_[TRAIT]_[TRANSFORMATION]_[SAMPLE]_[RACE]_chr[N]_[MMDDYYYY].Rdata where

TRAIT is either eGFRcrea, eGFRcys, or UACR;
TRANSFORMATION is either log or invnorm;
SAMPLE is either overall, DM, or nonDM;
RACE is either EA or AA;
N=1 to 22 and X for the chromosomes.

Each outcome, e.g. log(eGFRcrea), will have 23 files for each ethnic group (one for each chromosome). For file upload, please zip up the output for one outcome into two or three files.

Examples of file name for one study are given below. :

I. **European Americans (or Europeans):**
ARIC_ES_eGFRcrea_log_overall_EA_chr1_01202014.Rdata
ARIC_ES_eGFRcrea_invnorm_overall_EA_chr1_01202014.Rdata
ARIC_ES_eGFRcys_log_overall_EA_chr1_01202014.Rdata
ARIC_ES_eGFRcys_invnorm_overall_EA_chr1_01202014.Rdata
ARIC_ES_UACR_log_overall_EA_chr1_01202014.Rdata
ARIC_ES_UACR_invnorm_overall_EA_chr1_01202014.Rdata

II. **African Americans:**
ARIC_ES_eGFRcrea_log_overall_AA_chr1_01202014.Rdata
ARIC_ES_eGFRcrea_invnorm_overall_AA_chr1_01202014.Rdata
ARIC_ES_eGFRcys_log_overall_AA_chr1_01202014.Rdata
ARIC_ES_eGFRcys_invnorm_overall_AA_chr1_01202014.Rdata
ARIC_ES_UACR_log_overall_AA_chr1_01202014.Rdata
ARIC_ES_UACR_invnorm_overall_AA_chr1_01202014.Rdata

Step 8: Repeat the analysis for log eGFRcrea and UACR and inverse normal transformation of their residuals among those with diabetes

Examples of file name for eGFRcrea and UACR with diabetes are given below. :

I. **European Americans (or Europeans):**
ARIC_ES_eGFRcrea_log_DM_EA_chr1_01202014.Rdata
ARIC_ES_eGFRcrea_invnorm_DM_EA_chr1_01202014.Rdata
ARIC_ES_UACR_log_DM_EA_chr1_01202014.Rdata
ARIC_ES_UACR_invnorm_DM_EA_chr1_01202014.Rdata

II. **African Americans:**
ARIC_ES_eGFRcrea_log_DM_AA_chr1_01202014.Rdata
ARIC_ES_eGFRcrea_invnorm_DM_AA_chr1_01202014.Rdata
ARIC_ES_UACR_log_DM_AA_chr1_01202014.Rdata
ARIC_ES_UACR_invnorm_DM_AA_chr1_01202014.Rdata

Step 9: Repeat the analysis for log eGFRcrea and UACR and inverse normal transformation of their residuals among those without diabetes

Examples of file name for eGFRcrea and UACR without diabetes are given below. :

I. **European Americans (or Europeans):**
ARIC_ES_eGFRcrea_log_nonDM_EA_chr1_01202014.Rdata
ARIC_ES_eGFRcrea_invnorm_nonDM_EA_chr1_01202014.Rdata
ARIC_ES_UACR_log_nonDM_EA_chr1_01202014.Rdata
ARIC_ES_UACR_invnorm_nonDM_EA_chr1_01202014.Rdata

II. **African Americans:**
ARIC_ES_eGFRcrea_log_nonDM_AA_chr1_01202014.Rdata
ARIC_ES_eGFRcrea_invnorm_nonDM_AA_chr1_01202014.Rdata
ARIC_ES_UACR_log_nonDM_AA_chr1_01202014.Rdata
ARIC_ES_UACR_invnorm_nonDM_AA_chr1_01202014.Rdata

Example R code for the cohort level analysis in unrelated individuals:
Command 1: Load CHARGE SNP information file

```
snpinfo = get(load("snpinfo_WES_v13_Analytic_ChrN_10202015.Rdata"))
```

Command 2: Create a seqMeta object containing one cohort's contributions

```
# for log(eGFRcrea)
cohort_score = prepScores(Z = genos_chrN, formula = "eGFRcrea_log ~ age
+ factor(gender) + factor(center) + PC1", data = pheno, SNPInfo =
snpinfo, snpNames="SNP", aggregateBy = "SKATgene")

# for inverse normal transformed residuals of log(eGFRcrea)
cohort_score_2 = prepScores(Z = genos_chrN, formula =
"egfr_log_resid_inv ~1", data = pheno, SNPInfo = snpinfo,
snpNames="SNP", aggregateBy = "SKATgene")
```

Command 3: Save the result as Rdata file

```
save(cohort_score,file =
"COHORTA_ES_eGFRcrea_log_overall_EA_chrN_04042014.Rdata")
```

Example R code for cohort level analysis in related individuals:
Command 1: Load in the central SNP information fil

```
snpinfo = get(load("snpinfo_WES_v13_Analytic_ChrN_10202015.Rdata"))
```

Command 2: Create a seqMeta object containing one cohort's contributions

```
# for log(eGFRcrea)
cohort_score = prepScores(Z = genos_chrN,formula = "eGFRcrea_log ~ age
+ factor(gender) + factor(center) + PC1", data = pheno, SNPInfo =
snpinfo, fullkins = makekinship(fullped$fam, fullped$id, fullped$fa,
fullped$mo), snpNames="SNP", aggregateBy = "SKATgene")

# for inverse normal transformed residuals of log(eGFRcrea)
cohort_score = prepScores(Z = genos_chrN,formula = "egfr_log_resid_inv
~1", data = pheno, SNPInfo = snpinfo, fullkins =
makekinship(fullped$fam, fullped$id, fullped$fa, fullped$mo),
snpNames="SNP", aggregateBy = "SKATgene")
```

Command 3: Save the result as Rdata file

```
save(cohort_score, file =
'COHORTB_ES_eGFRcrea_log_overall_EA_chrN_04042014.Rdata')
```

After you have performed these analyses. Please fill in the attached tables and upload your results files <u>within 4 weeks</u> to the following Google drive:

"CHARGE Renal exome sequencing upload site" at
https://drive.google.com/drive/folders/0BzYDtCo_doHJRjlwZTFLajl4YXM

To access this Google drive:

1) Sign up for a Google Account ID (username@gmail.com) and password at: http://accounts.google.com/SignUp

2) Once they receive their ID, the ID and your study name should be emailed to Mira (chargeco@uw.edu) and you will be added to the access list (please cc Anna Kottgen (anna.koettgen@uniklinik-freiburg.de) and Adrienne Tin (atin1@jhu.edu)
Please also include the name of the Google drive folder you are requesting access to (CHARGE - Renal exome sequencing upload site). Once your ID has been added, you will receive an email from chargeco@uw.edu.

3) Please remember to email us the excel worksheet with your study sample information.

Any questions? Please contact Anna Kottgen (anna.koettgen@uniklinik-freiburg.de) and Adrienne Tin (atin1@jhu.edu)

Renal and Urate Traits Meta-analysis Plan using Genotypes from Whole Exome Sequencing
June 2016

1) Traits covered in this plan: both renal and urate traits:

Trait	Stratum	Transformation	Ancestry	Participating studies	Approx. sample size
eGFRcrea	overall	inverse normal, log	EA	ARIC, CHS, FHS, RS, ESP, ERF, CoLaus, Cilento	14363
eGFRcrea	DM	inverse normal, log	EA	ARIC, CHS, FHS, RS, ESP, ERF, CoLaus, Cilento	1355
eGFRcrea	nonDM	inverse normal, log	EA	ARIC, CHS, FHS, RS, ESP, ERF, CoLaus, Cilento	11530
eGFRcys	overall	inverse normal, log	EA	ARIC, CHS, FHS, RS, ESP	10859
UACR	overall	inverse normal, log	EA	ARIC, CHS, FHS, RS, ESP, CoLaus	11004
UACR	DM	inverse normal, log	EA	ARIC, CHS, FHS, RS, ESP, CoLaus	1003
UACR	nonDM	inverse normal, log	EA	ARIC, CHS, FHS, RS, ESP, CoLaus	8856
Gout	overall	N/A	EA	ARIC, CHS, FHS, RS, ESP	7416
Urate	overall	inverse normal	EA	ARIC, CHS, FHS, RS, ESP, CoLaus	11896
eGFRcrea	overall	inverse normal, log	AA	ARIC, ESP	4080
eGFRcrea	DM	inverse normal, log	AA	ARIC	479
eGFRcrea	nonDM	inverse normal, log	AA	ARIC	2339
eGFRcys	overall	inverse normal, log	AA	ARIC	1872
UACR	overall	inverse normal, log	AA	ARIC, ESP	1901
UACR	DM	inverse normal, log	AA	ARIC	270
UACR	nonDM	inverse normal, log	AA	ARIC	1627
Gout	overall	N/A	AA	ARIC	1979
Urate	overall	inverse normal	AA	ARIC	3626

2) Primary analyses
   A) Regarding transformations, the analyses using inverse normal transformation are our primary analyses for determining statistical significance. The analyses using the log transformed outcomes are mainly for obtaining beta estimates for comparison with published results.
   B) Overall vs stratified analysis. We will use the overall analyses as our primary analyses. The number of variants and genes in the overall analysis will be used for setting the significance threshold.
   C) Gene-based tests. Section 4B lists 4 gene-based tests. The primary analyses are the test including MAF < 1% and MAF < 5%.

3) Filters that are applicable to all tests:
   A) Single variant test filters:

i) Variant annotation filter: none

ii) Minor allele count filter: 10 will likely yield a qq-plot without showing p-value inflation and a genomic control that is close to 1 using the inverse normal transformed traits. We may adapt this threshold based on the qq-plot of the meta-analysis.

B) Gene-based test filters:

i) Variant annotation filters
We will include the union of the following 3 sets of variants: a) nonsynonymous and splice sites as defined by sc_nonsynSplice; b) predicted splice sites defined by splicing_consensus_ada_score ≥ 0.6, splicing_consensus_rf_score ≥ 0.6, or absolute value of SPIDEX_dpsi_max_tissue > 5; c) coding indels defined by sc_indel_coding.

ii) Gene filter: contain 2 or more variants in meta-analysis

iii) Cumulative MAF filters: requiring at least 10 copies in a gene. We may adapt this threshold based on the GC parameter and qq-plot of the meta-analysis.

C) Gene-based test weight

i) beta distribution of MAF with parameters alpha=1, beta=25 unless specified otherwise below.

4) Meta-analysis within EA, AA, and combined EA and AA populations

A) Single variant test

B) Gene-based tests

i) Two primary SKAT-O tests: 1) MAF < 1%, 2) MAF < 5%

ii) Two secondary SKAT-O tests: 1) no limit on MAF weighted by beta distribution, 2) no limit on MAF weighted by CADD phred score (aka CADD scaled score). Variants without CADD phred scores will be assigned a score that is the median of variants in sc_nonsynSplice by MAF bin (<0.1%, ≥ 0.1% to <1%, ≥1% to <5%, and ≥5% based on MAF in CHARGE Freeze 5).

5) Statistical significance threshold

A) Single variant test:

i) Kidney function traits: 0.05/(number of variants after MAC filter in the EA and AA combined overall analysis of eGFRcrea)

ii) Serum urate: 0.05/(number of variants after MAC filter in the EA and AA combined overall analysis of serum urate)

B) Gene-based tests:

i) Kidney function traits: 0.05/(number of genes * number of primary tests in the EA and AA combined analysis of eGFRcrea)

ii) Serum urate: 0.05/(number of genes * number of primary tests in the EA and AA combined analysis of serum urate)

Renal and Urate Exome Sequencing Project
Interrogation of Susceptibility Loci Analysis Plan (October 2016)

<u>Overview of Interrogation Analysis</u>

The purpose of these analyses is to evaluate whether known kidney or uric acid susceptibility variants or genes are associated with the corresponding traits in the exome sequencing meta-analysis results.

Sources of known kidney or uric acid susceptibility variants or genes will be retrieved from the following data sources with details described in the sections below on the specific traits:

1) GWAS catalog
2) Mendelian genes: published lists of genes causing Mendelian forms of the disease in human
3) Mouse model genes: genes associated with corresponding traits in mouse models in the Mouse Genome Informatics (MGI) databases.

Targets of queries will be the results of the primary analyses in EA+AA of the corresponding traits, which will be used for determining statistical significance. The results from EA and AA will also be retrieved to inform heterogeneity across ethnic groups. The primary analyses in EA+AA are:

1) Single variant meta-analysis filtered by ≥ 10 copies of the minor allele
2) SKATO meta-analysis of putative functional variants with MAF < 1%
3) SKATO meta-analysis of putative functional variants with MAF < 5%

The tables generated from this analysis plan should contain all information necessary for publication plus other useful columns that may be trimmed later (see spreadsheet).

Definition of query regions and significance threshold

1) **GWAS catalog**. The query region will be defined as 500kb on both sides of the index SNP in the GWAS catalog. Recombination hot spots are not considered here because significant index SNPs in GWAS tend to lie in regulatory regions. The causal regulating SNP and the target gene may be separate by recombination hot spot. If two index SNPs are less than 500kb apart, then the query region will be defined as 500kb around the middle of the two index SNPs.
   A) **Single variant query significant threshold**. Since exonic variants tend to have low LD, the p-value significant threshold will be defined as 0.05 divided by the number of variants with ≥ 10 copies of the minor allele in the query region in the EA+AA meta-analysis.
   B) **Gene-based test query significant threshold**. 0.05 divided by the number of genes with ≥ 2 variants in the query region * 2 for two SKATO tests in EA+AA meta-analysis.

2) **Mendelian genes**. The query region and significant threshold are defined similar to the procedure in Jing et al. KI, 2016 with adaptation for exonic variants. The query region will be defined as 10kb upstream or downstream of the gene.
   A) **Single variant query significant threshold**: 0.05 divided by the **total** number of **index variants** across **all** genes in the corresponding trait. An **index variant** of a gene is defined as the variant with the lowest p-value in the gene region and a minor allele count ≥ 10 copies.
   B) **Gene-based test query significant threshold**: 0.05 divided by the total number of genes with ≥ 2 variants in our meta-analysis of the corresponding trait * 2 for two SKATO tests in EA+AA.
3) **Mouse model genes**. The query region and the significant threshold will be defined as the same as in the query using the Mendelian genes.

__Specifics for kidney traits (eGFR and UACR)__

Criteria for selecting susceptibility variants or genes for kidney traits (eGFR and UACR)

	Criteria
GWAS catalog	1) Index SNPs of the following DISEASE.TRAIT:<br>• Chronic kidney disease<br>• Chronic kidney disease and serum creatinine levels<br>• Glomerular filtration rate<br>• Glomerular filtration rate in chronic kidney disease<br>• Glomerulosclerosis<br>• IgA nephropathy<br>• Kidney function decline traits<br>• Nephrotic syndrome (acquired)<br>• Proteinuria and chronic kidney disease<br>• Proteinuria in chronic kidney disease<br>• Renal function and chronic kidney disease<br>• Renal function-related traits (BUN)<br>• Renal function-related traits (eGRFcrea)<br>• Renal function-related traits (sCR)<br>• Renal function-related traits (urea)<br>2) GWAS catalog p-value < 5e-8<br>3) Loci discovered in adult populations
Mendelian genes	The panel in Larsen et al. AJKD, 2016 contains 301 genes. Our queries will include the 265 clinical genes that have known association with nephropathies, but not the 36 “experimental” genes (see spreadsheet)
Mouse model genes	The selection of renal susceptibility genes from the MGI database will follow the same procedure as described in Jing et al. KI, 2016. Briefly, the following

	phenotype from the **Mammalian Phenotype Ontology** will be used to identify susceptibility genes for renal traits in mouse:<br>• MP: 0002847, abnormal GFR<br>• MP: 0002136, abnormal kidney physiology<br>• MP: 0002135, abnormal kidney morphology<br>• MP: 0006315, abnormal urine protein level<br>The corresponding gene in human will be identified using the **Human-Mouse:Disease Connection** database

Targets of queries

All queries for the renal traits will include eGFRcrea overall and UACR overall meta-analysis results in EA+AA, EA, and AA in one table because in the above data sources some phenotypes are associated with both GFR and proteinuria.
The **single variant query** will include the results from the eGFRcrea inverse normal overall meta-analysis in EA+AA, EA, and AA followed by the results from the UACR inverse normal overall meta-analysis in EA+AA, EA, and AA.
The **SKATO query** will include the results from the eGFRcrea inverse normal overall meta-analysis for MAF < 1% in EA+AA, EA, and AA followed by the results from MAF < 5%, then the results from the UACR inverse normal overall meta-analysis for MAF < 1% in EA+AA, EA, and AA followed by the results from MAF < 5%,

<u>**Specifics for urate traits (serum urate and gout)**</u>

Criteria for selecting susceptibility variants or genes for urate traits (urate and gout)

	Criteria
GWAS catalog	1) Index SNPs with the following values in the DISEASE.TRAIT variable:<br>• Gout<br>• Serum uric acid levels<br>• Urate levels<br>• Urate levels (BMI interaction)<br>• Urate levels in lean individuals<br>• Urate levels in obese individuals<br>• Urate levels in overweight individuals<br>• Uric acid levels<br>2) GWAS catalog p-value < 5e-8
Mendelian genes	OMIM Advanced Search: select **MIM number Prefix**: "# phenotype description, molecular basis known" with the keyword **hyperuricemia** or **hypouricemia**. After the list of phenotypes are displayed, retrieve corresponding **Gene Map**

Mouse model genes	The selection of urate susceptibility genes from the MGI database will follow the procedure as described in Jing et al. KI, 2016 and adapted for urate. Briefly, the following phenotype term in the **Mammalian Phenotype Ontology** will be used to identify susceptibility genes for urate traits in mouse:<br>• MP: 0008820, abnormal blood uric acid level<br>MP: 0008820 includes:<br>• MP: 0008822, decreased blood uric acid level<br>• MP: 0008821, increased blood uric acid level<br>The corresponding genes in human will be identified using the **Human-Mouse:Disease Connection** database

Targets of queries

All queries for the urate trait will include serum urate and gout meta-analysis results in EA+AA, EA, and AA in one table (see table shell example)
The **single variant query** will include the results from serum urate inverse normal meta-analysis in EA+AA, EA, and AA followed by the results from gout meta-analysis in EA+AA, EA, and AA.
The **SKATO query** will include the results from serum urate inverse normal overall meta-analysis for MAF < 1% in EA+AA, EA, and AA followed by the results from MAF < 5%, then the results from gout meta-analysis for MAF < 1% in EA+AA, EA, and AA followed by the results from MAF < 5%.

References

A Custom Targeted Next-Generation Sequencing Gene Panel for the Diagnosis of Genetic Nephropathies. Larsen CP, Durfee T, Wilson JD, Beggs ML. Am J Kidney Dis. 2016 Jun;67(6):992-3.

Combination of mouse models and genomewide association studies highlights novel genes associated with human kidney function. Jing J, Pattero C, Hoppmann A, et al. Kidney International (2016) 90, 764–773;

Replication of Association between rs150255373 at SLC22A12 (Chr 11, 64366298 GRCh37 position, C ->T) and Systolic Blood Pressure (SBP): Analysis Plan

Background

This low frequency variant, T allele, was identified as associated with lower serum urate levels in a multi-cohort exome-wide association study. To follow-up on the long-standing controversy on the relation between serum urate and blood pressure, we found that this variant was also associated with lower blood pressure and wish to replicate this association in additional cohorts.

Outcome Definition and Covariates

The following definition and covariates are the same as in Yu et al. Circ Cardiovasc Genet, 2016

Systolic blood pressure (SBP) definition

1) For individuals under hypertension medication treatment, add 15 mmHg
2) Exclude individuals with untreated SBP < 60 mmHg
3) Winsorize the trait at 99.9^{th} percentile

Covariates: age, age-squared, sex, BMI, genetic principal components, study site if applicable.

Analysis

Please use linear regression and the HRC imputed dosage as predictor of interest. Please carry out the analyses overall, as well as separately in men and women (not including sex as a covariate). If the number of carriers of the T allele is < 5, please provide the unadjusted SBP and hypertension medication status of each carrier of the T allele in the **Readme** spreadsheet.

For our internal information, additional information on associations with blood urate concentrations have been looked up from the urate summary statistics submitted to the CKDGen R4 analyses. The results of your study from this data submission have been prefilled in the **Reg_results** spreadsheet. To gain more insight into this variant, we appreciate if you could provide the additional information in columns M to U in the **Reg_results** sheet for the urate results. In case an interesting observation with SBP is observed, we may be in touch with an official lookup request for this variant on the urate association.

Results

Please provide the results in the attached spreadsheet.

The following table provides a brief description of the columns in the spreadsheet

Columns in the regression result spreadsheet (**Reg_results**):

Variable	Description
Beta	Beta coefficient for each copy of the T allele
SE	Standard error
p-value	p-value of rs150255373
MAF	Minor allele frequency
MAC	Minor allele count

ref	Non-coding allele
alt	Coding allele
N_total	Analyzed sample size
Imp_qual	Mean imputation quality
Num_CT	If the data is imputed, please use 0.5 to 1.5 to convert imputed dosage to imputed genotype. If your study already has a preferred method for conversion to most likely genotype, please use your study's method and note the method.
Num_TT	If the data is imputed and C is the reference, please use dosage > 1.5 to convert imputed dosage to imputed genotype. If your study already has a preferred method for conversion to most likely genotype, please use your study's method and note the method.
Num_urate_med	Number of participants on urate lowering medications if available
Urate_med	If urate lowering medication is available, please note the kind of urate lowering medication included, e.g. allopurino
SBP_mean	Mean SBP of the analyzed sample
SBP_SD	Standard deviation of the SBP in the analyzed sample
Male_pct	Percentage of male participants overall
HT_med_pct	Percentage of participants who were on hypertension medication overall
Carrier_male	Number of carriers of the T allele who are male based on imputed genotype
Carrier_HT_med	Number of carriers of the T allele who were on hypertensive medications based on imputed genotype

Please provide the following information in the **Readme** spreadsheet

Information	Description
Genotype_method	Method for converting imputed dosage to imputed genotype
Genetic_PC_criteria	Criteria for selecting genetic PCs, e.g. first 10 or those significantly associated with SBP among the first 10.

Please address inquiries on this analysis to Adrienne Tin (atin1@jhu.edu) and Anna Kottgen (anna.koettgen@uniklinik-freiburg.de)

Supplementary Note 3 Acknowledgements

The Atherosclerosis Risk in Communities (ARIC) Study is carried out as a collaborative study supported by National Heart, Lung, and Blood Institute contracts (HHSN268201100005C, HHSN268201100006C, HHSN268201100007C, HHSN268201100008C, HHSN268201100009C, HHSN268201100010C, HHSN268201100011C and HHSN268201100012C), R01HL087641, R01HL59367 and R01HL086694; National Human Genome Research Institute contract U01HG004402; and National Institutes of Health contract HHSN268200625226C. Infrastructure was partly supported by Grant Number UL1RR025005, a component of the National Institutes of Health and NIH Roadmap for Medical Research. The authors thank the staff and participants of the ARIC study for their important contributions. Funding. This study was supported by the National Institute of Diabetes and Digestive and Kidney Diseases R01 DK076770–01.

The Cardiovascular Health Study (CHS) was supported by NHLBI contracts HHSN268201200036C, HHSN268200800007C, N01HC55222, N01HC85079, N01HC85080, N01HC85081, N01HC85082, N01HC85083, N01HC85086; and NHLBI grants U01HL080295, R01HL087652, R01HL105756, R01HL103612, R01HL120393, and R01HL130114 with additional contribution from the National Institute of Neurological Disorders and Stroke (NINDS). Additional support was provided through R01AG023629 from the National Institute on Aging (NIA). A full list of principal CHS investigators and institutions can be found at CHS-NHLBI.org. The provision of genotyping data was supported in part by the National Center for Advancing Translational Sciences, CTSI grant UL1TR000124, and the National Institute of Diabetes and Digestive and Kidney Disease Diabetes Research Center (DRC) grant DK063491 to the Southern California Diabetes Endocrinology Research Center. The content is solely the responsibility of the authors and does not necessarily represent the official views of the National Institutes of Health.

The Cilento study was supported by grants from the Italian Ministry of Universities and Research and CNR (Interomics Flagship Project, PON03PE_00060_7), the Assessorato Ricerca Regione Campania, the Fondazione con il SUD (2011-PDR-13), and the Istituto Banco di Napoli - Fondazione to MC. We thank the populations of Cilento for their participation in the study.

The CoLaus study was and is supported by research grants from GlaxoSmithKline, the Faculty of Biology and Medicine of Lausanne, and the Swiss National Science Foundation (grants 33CSCO-122661, 33CS30-139468 and 33CS30-148401). The authors thank Gerard Waeber, Vincent Mooser and Dawn Waterworth, Co-PIs of the CoLaus study and all the participants of the study. SB is supported by the Swiss National Science Foundation (grant 3100AO-116323/1) and the Swiss Institute of Bioinformatics. ZK received financial support from the Leenaards Foundation, the Swiss Institute of Bioinformatics and the

Swiss National Science Foundation (31003A-169929) and SystemsX.ch (51RTP0_151019).

The Erasmus Rucphen Family (ERF) study as a part of EUROSPAN (European Special Populations Research Network) was supported by European Commission FP6 STRP grant number 018947 (LSHG-CT2006-01947) and also received funding from the European Community's Seventh Framework Programme (FP7/2007-2013)/grant agreement HEALTH-F4-2007-201413 by the European Commission under the programme "Quality of Life and Management of the Living Resources" of 5th Framework Programme (no. QLG2-CT-2002-01254). Highthroughput analysis of the ERF data was supported by joint grant from Netherlands Organization for Scientific Research and the Russian Foundation for Basic Research (NWO-RFBR 047.017.043). Exome sequencing analysis in ERF was supported by the ZonMw grant (project 91111025). Ayşe Demirkan is supported by a Veni grant (2015) from ZonMw. Ayşe Demirkan, Jun Liu and Cornelia van Duijn have used exchange grants from Personalized pREvention of Chronic DIseases consortium (PRECeDI) (H2020-MSCA-RISE-2014). We are grateful to all study participants and their relatives, general practitioners and neurologists for their contributions and to P. Veraart for her help in genealogy, J. Vergeer for the supervision of the laboratory work and P. Snijders for his help in data collection.

Funding for GO ESP was provided by NHLBI grants RC2 HL-103010 (HeartGO), RC2 HL-102923 (LungGO) and RC2 HL-102924 (WHISP). The exome sequencing was performed through NHLBI grants RC2 HL-102925 (BroadGO) and RC2 HL-102926 (SeattleGO). The authors wish to acknowledge the support of the National Heart, Lung, and Blood Institute (NHLBI) and the contributions of the research institutions, study investigators, field staff and study participants in creating this resource for biomedical research.

The Framingham Heart Study is conducted and supported by the NHLBI in collaboration with Boston University (Contract No. N01-HC-25195 and HHSN268201500001I), and its contract with Affymetrix, Inc., for genome-wide genotyping services (Contract No. N02-HL-6-4278), for quality control by Framingham Heart Study investigators using genotypes in the SNP Health Association Resource (SHARe) project. A portion of this research was conducted using the Linux Cluster for Genetic Analysis (LinGA) computing resources at Boston University Medical Campus. This study is part of the NHLBI Grand Opportunity Exome Sequencing Project (GO-ESP).

The Rotterdam Study (RS) is funded by Erasmus Medical Center and Erasmus University, Rotterdam, Netherlands Organization for the Health Research and Development (ZonMw), the Research Institute for Diseases in the Elderly (RIDE), the Ministry of Education, Culture and Science, the Ministry for Health, Welfare and Sports, the European Commission (DG XII), and the Municipality of Rotterdam. The authors are grateful to the study participants, the staff from the Rotterdam Study and the participating general practitioners and pharmacists. The generation and management of the exome sequencing data for the Rotterdam Study was executed by the Human Genotyping Facility of the

Genetic Laboratory of the Department of Internal Medicine, Erasmus MC, the Netherlands. The Exome Sequencing data set was funded by the Netherlands Genomics Initiative (NGI)/Netherlands Organisation for Scientific Research (NWO) sponsored Netherlands Consortium for Healthy Aging (NCHA; project nr. 050-060-810), by the Genetic Laboratory of the Department of Internal Medicine, Erasmus MC, and by the and by a Complementation Project of the Biobanking and Biomolecular Research Infrastructure Netherlands (BBMRI-NL; www.bbmri.nl; project number CP2010-41). We thank Mr. Pascal Arp, Ms. Mila Jhamai, Jeroen van Rooij, MSc, Mr. Marijn Verkerk, and Robert Kraaij, PhD for their help in creating the RS-Exome Sequencing database.

SHIP is part of the Community Medicine Research net of the University of Greifswald, Germany, which is funded by the Federal Ministry of Education and Research (grants no. 01ZZ9603, 01ZZ0103, and 01ZZ0403), the Ministry of Cultural Affairs as well as the Social Ministry of the Federal State of Mecklenburg-West Pomerania, and the network 'Greifswald Approach to Individualized Medicine (GANI_MED)' funded by the Federal Ministry of Education and Research (grant 03IS2061A). Generation of ExomeChip data was supported by the Federal Ministry of Education and Research (grant no. 03Z1CN22). The University of Greifswald is a member of the Caché Campus program of the InterSystems GmbH.

NHLBI GO Exome Sequencing Project

Broad GO:

Stacey B. Gabriel (Broad Institute)[4, 5, 11, 16, 17], David M. Altshuler (Broad Institute, Harvard Medical School, Massachusetts General Hospital)[1,5,7,17], Gonçalo R. Abecasis (University of Michigan)[3, 5, 9, 13, 15, 17], Hooman Allayee (University of Southern California)[5], Sharon Cresci (Washington University School of Medicine)[5], Mark J. Daly (Broad Institute, Massachusetts General Hospital), Paul I. W. de Bakker (Broad Institute, Harvard Medical School, University Medical Center Utrecht)[3, 15], Mark A. DePristo (Broad Institute)[4, 13, 15, 16], Ron Do (Broad Institute)[5, 9, 13, 15], Peter Donnelly (University of Oxford)[5], Deborah N. Farlow (Broad Institute)[3, 4, 5, 12, 14, 16, 17], Tim Fennell (Broad Institute), Kiran Garimella (University of Oxford)[4, 16], Stanley L. Hazen (Cleveland Clinic)[5], Youna Hu (University of Michigan)[3, 9, 15], Daniel M. Jordan (Harvard Medical School, Harvard University)[13], Goo Jun (University of Michigan)[13], Sekar Kathiresan (Broad Institute, Harvard Medical School, Massachusetts General Hospital)[5, 8, 9, 12, 14, 15, 17, 20], Hyun Min Kang (University of Michigan)[9, 13, 16], Adam Kiezun (Broad Institute)[5, 13, 15], Guillaume Lettre (Broad Institute, Montreal Heart Institute, Université de Montréal)[1, 2, 13, 15], Bingshan Li (University of Michigan)[3], Mingyao Li (University of Pennsylvania)[5], Christopher H. Newton-Cheh (Broad Institute, Massachusetts General Hospital, Harvard Medical School)[3, 8, 15], Sandosh Padmanabhan (University of Glasgow School of Medicine)[3, 12, 15], Gina Peloso (Broad Institute, Harvard Medical School, Massachusetts General Hospital)[5], Sara Pulit (Broad Institute)[3, 15], Daniel J. Rader (University of Pennsylvania)[5], David Reich (Broad Institute, Harvard Medical School)[15], Muredach P. Reilly (University of Pennsylvania)[5], Manuel A. Rivas (Broad Institute, Massachusetts General Hospital)[5], Steve Schwartz (Fred Hutchinson Cancer

Research Center)[5, 12], Laura Scott (University of Michigan)[1], David S. Siscovick (University of Washington)[5, 1, 25], John A. Spertus (University of Missouri Kansas City)[5], Nathaniel O. Stitziel (Brigham and Women's Hospital)[5, 15], Nina Stoletzki (Brigham and Women's Hospital, Broad Institute, Harvard Medical School)[13], Shamil R. Sunyaev (Brigham and Women's Hospital, Broad Institute, Harvard Medical School)[1, 3, 5, 13, 15], Benjamin F. Voight (Broad Institute, Massachusetts General Hospital), Cristen J. Willer (University of Michigan)[1, 9, 13, 15].

Heart GO:

Stephen S. Rich (University of Virginia)[2, 4, 7, 8, 9, 11,14, 15, 17, 18, 31], Ermeg Akylbekova (Jackson State University, University of Mississippi Medical Center)[29], Larry D. Atwood (Boston University)[1, 11, 28], Christie M. Ballantyne (Baylor College of Medicine, Methodist DeBakey Heart Center)[9, 22], Maja Barbalic (University of Texas Health Science Center Houston)[9, 14, 15, 17, 22], R. Graham Barr (Columbia University Medical Center)[10, 31], Emelia J. Benjamin (Boston University)[14, 20, 28], Joshua Bis (University of Washington)[15, 23], Eric Boerwinkle (University of Texas Health Science Center Houston)[3, 5, 9, 13, 15, 17, 22], Donald W. Bowden (Wake Forest University)[1, 31], Jennifer Brody (University of Washington)[3, 5, 15, 23], Matthew Budoff (Harbor-UCLA Medical Center)[31], Greg Burke (Wake Forest University)[5, 31], Sarah Buxbaum (Jackson State University)[3, 13, 15, 29], Jeff Carr (Wake Forest University)[25, 29, 31], Donna T. Chen (University of Virginia)[6, 11], Ida Y. Chen (Cedars-Sinai Medical Center)[1, 31], Wei-Min Chen (University of Virginia)[13, 15, 18], Pat Concannon (University of Virginia)[11], Jacy Crosby (University of Texas Health Science Center Houston)[22], L. Adrienne Cupples (Boston University)[1, 3, 5, 9, 13, 15, 18, 28], Ralph D'Agostino (Boston University)[28], Anita L. DeStefano (Boston University)[13, 18, 28], Albert Dreisbach (University of Mississippi Medical Center)[3, 29], Josée Dupuis (Boston University)[1, 28], J. Peter Durda (University of Vermont)[15, 23], Jaclyn Ellis (University of North Carolina Chapel Hill)[1], Aaron R. Folsom (University of Minnesota)[5, 22], Myriam Fornage (University of Texas Health Science Center Houston)[3, 18, 25], Caroline S. Fox (National Heart, Lung, and Blood Institute)[1, 28], Ervin Fox (University of Mississippi Medical Center)[3, 9, 29], Vincent Funari (Cedars-Sinai Medical Center)[1, 11, 31], Santhi K. Ganesh (University of Michigan)[2, 22], Julius Gardin (Hackensack University Medical Center)[25], David Goff (Wake Forest University)[25], Ora Gordon (Cedars-Sinai Medical Center)[11, 31], Wayne Grody (University of California Los Angeles)[11, 31], Myron Gross (University of Minnesota)[1, 5, 14, 25], Xiuqing Guo (Cedars-Sinai Medical Center)[3, 15, 31], Ira M. Hall (University of Virginia), Nancy L. Heard-Costa (Boston University)[1, 11, 28], Susan R. Heckbert (University of Washington)[10, 14, 20, 23], Nicholas Heintz (University of Vermont), David M. Herrington (Wake Forest University)[5, 31], DeMarc Hickson (Jackson State University, University of Mississippi Medical Center)[29], Jie Huang (National Heart, Lung, and Blood Institute)[5, 28], Shih-Jen Hwang (Boston University, National Heart, Lung, and Blood Institute)[3, 28], David R. Jacobs (University of Minnesota)[25], Nancy S. Jenny (University of Vermont)[1, 2, 23], Andrew D. Johnson (National Heart, Lung, and Blood Institute)[2, 5, 11, 28], Craig W. Johnson (University of Washington)[15, 31], Steven Kawut (University of Pennsylvania)[10,31], Richard Kronmal (University of Washington)[31], Raluca Kurz (Cedars-Sinai Medical Center)[11, 31], Ethan M. Lange (University of North Carolina Chapel Hill)[3, 5, 9, 13, 34], Leslie A. Lange (University of North Carolina Chapel Hill)[1, 2, 3, 5, 9, 12,

[13, 15, 17, 18, 20, 25, 34], Martin G. Larson (Boston University)[3, 15, 28], Mark Lawson (University of Virginia), Cora E. Lewis (University of Alabama at Birmingham)[25,34], Daniel Levy (National Heart, Lung, and Blood Institute)[3, 15, 17, 28], Dalin Li (Cedars-Sinai Medical Center)[11, 15, 31], Honghuang Lin (Boston University)[20, 28], Chunyu Liu (National Heart, Lung, and Blood Institute)[3, 28], Jiankang Liu (University of Mississippi Medical Center)[1, 29], Kiang Liu (Northwestern University)[25], Xiaoming Liu (University of Texas Health Science Center Houston)[15, 22], Yongmei Liu (Wake Forest University)[2, 5, 31], William T. Longstreth (University of Washington)[18, 23], Cay Loria (National Heart, Lung, and Blood Institute)[25], Thomas Lumley (University of Auckland)[9, 23], Kathryn Lunetta (Boston University)[28], Aaron J. Mackey (University of Virginia)[16, 18], Rachel Mackey (University of Pittsburgh)[1, 23, 31], Ani Manichaikul (University of Virginia)[8, 15, 18, 31], Taylor Maxwell (University of Texas Health Science Center Houston)[22], Barbara McKnight (University of Washington)[15, 23], James B. Meigs (Brigham and Women's Hospital, Harvard Medical School, Massachusetts General Hospital)[1, 28], Alanna C. Morrison (University of Texas Health Science Center Houston)[3, 15, 17], Solomon K. Musani (University of Mississippi Medical Center)[3, 29], Josyf C. Mychaleckyj (University of Virginia)[13, 15, 31], Jennifer A. Nettleton (University of Texas Health Science Center Houston)[9, 22], Kari North (University of North Carolina Chapel Hill)[1, 3, 9, 10, 13, 15, 17, 34], Christopher J. O'Donnell (Massachusetts General Hospital, National Heart, Lung, and Blood Institute)[2, 5, 9, 11, 12, 14, 15, 17, 20, 28], Daniel O'Leary (Tufts University School of Medicine)[25, 31], Frank Ong (Cedars-Sinai Medical Center)[3, 11, 31], Walter Palmas (Columbia University)[3, 15, 31], James S. Pankow (University of Minnesota)[1, 22], Nathan D. Pankratz (Indiana University School of Medicine)[15, 25], Shom Paul (University of Virginia), Marco Perez (Stanford University School of Medicine), Sharina D. Person (University of Alabama at Birmingham, University of Alabama at Tuscaloosa)[25], Joseph Polak (Tufts University School of Medicine)[31], Wendy S. Post (Johns Hopkins University)[3, 9, 11, 14, 20, 31], Bruce M. Psaty (Group Health Research Institute, University of Washington)[3, 5, 9, 11, 14, 15, 23], Aaron R. Quinlan (University of Virginia)[18, 19], Leslie J. Raffel (Cedars-Sinai Medical Center)[6, 11, 31], Vasan S. Ramachandran (Boston University)[3, 28], Alexander P. Reiner (Fred Hutchinson Cancer Research Center, University of Washington)[1, 2, 3, 5, 9, 11, 12, 13, 14, 15, 20, 25, 34], Kenneth Rice (University of Washington)[15, 23], Jerome I. Rotter (Cedars-Sinai Medical Center)[1, 3, 6, 8, 11, 15, 31], Jill P. Sanders (University of Vermont)[23], Pamela Schreiner (University of Minnesota)[25], Sudha Seshadri (Boston University)[18, 28], Steve Shea (Brigham and Women's Hospital, Harvard University)[28], Stephen Sidney (Kaiser Permanente Division of Research, Oakland, CA)[25], Kevin Silverstein (University of Minnesota)[25], David S. Siscovick (University of Washington)[5, 1, 25], Nicholas L. Smith (University of Washington)[2, 15, 20, 23], Nona Sotoodehnia (University of Washington)[3, 15, 23], Asoke Srinivasan (Tougaloo College)[29], Herman A. Taylor (Jackson State University, Tougaloo College, University of Mississippi Medical Center)[5, 29], Kent Taylor (Cedars-Sinai Medical Center)[31], Fridtjof Thomas (University of Texas Health Science Center Houston)[3, 22], Russell P. Tracy (University of Vermont)[5, 9, 11, 12, 14, 15, 17, 20, 23], Michael Y. Tsai (University of Minnesota)[9, 31], Kelly A. Volcik (University of Texas Health Science Center Houston)[22], Chrstina L Wassel (University of California San Diego)[9, 15, 31], Karol Watson (University of California Los Angeles)[31], Gina Wei (National Heart, Lung, and Blood Institute)[25], Wendy White (Tougaloo College)[29], Kerri L. Wiggins (University of

Vermont)[23], Jemma B. Wilk (Boston University)[10, 28], O. Dale Williams (Florida International University)[25], Gregory Wilson (Jackson State University)[29], James G. Wilson (University of Mississippi Medical Center)[1, 2, 5, 8, 9, 11, 12, 14, 17, 20, 29], Phillip Wolf (Boston University)[28], Neil A. Zakai (University of Vermont)[2, 23].

ISGS and SWISS:

John Hardy (Reta Lila Weston Research Laboratories, Institute of Neurology, University College London)[18], James F. Meschia (Mayo Clinic)[18], Michael Nalls (National Institute on Aging)[2, 18], Stephen S. Rich (University of Virginia)[2, 4, 7, 8, 9, 11, 14, 15, 17, 18, 31], Andrew Singleton (National Institute on Aging)[18], Brad Worrall (University of Virginia)[18].

Lung GO:

Michael J. Bamshad (Seattle Children's Hospital, University of Washington)[4, 6, 7, 8, 10, 11, 13, 15, 17, 27], Kathleen C. Barnes (Johns Hopkins University)[2, 10, 12, 14, 15, 17, 20, 24, 30, 32], Ibrahim Abdulhamid (Children's Hospital of Michigan)[27], Frank Accurso (University of Colorado)[27], Ran Anbar (Upstate Medical University)[27], Terri Beaty (Johns Hopkins University)[24, 30], Abigail Bigham (University of Washington)[13, 15, 27], Phillip Black (Children's Mercy Hospital)[27], Eugene Bleecker (Wake Forest University)[33], Kati Buckingham (University of Washington)[27], Anne Marie Cairns (Maine Medical Center)[27], Wei-Min Chen (University of Virginia)[13, 15, 18], Daniel Caplan (Emory University)[27], Barbara Chatfield (University of Utah)[27], Aaron Chidekel (A.I. Dupont Institute Medical Center)[27], Michael Cho (Brigham and Women's Hospital, Harvard Medical School)[13, 15, 24], David C. Christiani (Massachusetts General Hospital)[21], James D. Crapo (National Jewish Health)[24, 30], Julia Crouch (Seattle Children's Hospital)6, Denise Daley (University of British Columbia)[30], Anthony Dang (University of North Carolina Chapel Hill)[26], Hong Dang (University of North Carolina Chapel Hill)[26], Alicia De Paula (Ochsner Health System)[27], Joan DeCelie-Germana (Schneider Children's Hospital)[27], Allen Dozor (New York Medical College, Westchester Medical Center)[27], Mitch Drumm (University of North Carolina Chapel Hill)[26], Maynard Dyson (Cook Children's Med. Center)[27], Julia Emerson (Seattle Children's Hospital, University of Washington)[27], Mary J. Emond (University of Washington)[10, 13, 15, 17, 27], Thomas Ferkol (St. Louis Children's Hospital, Washington University School of Medicine)[27], Robert Fink (Children's Medical Center of Dayton)[27], Cassandra Foster (Johns Hopkins University)[30], Deborah Froh (University of Virginia)[27], Li Gao (Johns Hopkins University)[24, 30, 32], William Gershan (Children's Hospital of Wisconsin)[27], Ronald L. Gibson (Seattle Children's Hospital, University of Washington)[10, 27], Elizabeth Godwin (University of North Carolina Chapel Hill)[26], Magdalen Gondor (All Children's Hospital Cystic Fibrosis Center)[27], Hector Gutierrez (University of Alabama at Birmingham)[27], Nadia N. Hansel (Johns Hopkins University, Johns Hopkins University School of Public Health)[10, 15, 30], Paul M. Hassoun (Johns Hopkins University)[10, 14, 32], Peter Hiatt (Texas Children's Hospital)[27], John E. Hokanson (University of Colorado)[24], Michelle Howenstine (Indiana University, Riley Hospital for Children)[27], Laura K. Hummer (Johns Hopkins University)[32], Jamshed Kanga (University of Kentucky)[27], Yoonhee Kim (National Human Genome Research Institute)[24, 32], Michael R. Knowles (University of North Carolina Chapel Hill)[10, 26], Michael Konstan (Rainbow Babies & Children's Hospital)[27], Thomas Lahiri (Vermont Children's Hospital at Fletcher Allen Health Care)[27], Nan Laird (Harvard School

of Public Health)[24], Christoph Lange (Harvard School of Public Health)[24], Lin Lin (Harvard Medical School)[21], Xihong Lin (Harvard School of Public Health)[21], Tin L. Louie (University of Washington)[13, 15, 27], David Lynch (National Jewish Health)[24], Barry Make (National Jewish Health)[24], Thomas R. Martin (University of Washington, VA Puget Sound Medical Center)[10, 21], Steve C. Mathai (Johns Hopkins University)[32], Rasika A. Mathias (Johns Hopkins University)[10, 13, 15, 30, 32], John McNamara (Children's Hospitals and Clinics of Minnesota)[27], Sharon McNamara (Seattle Children's Hospital)[27], Deborah Meyers (Wake Forest University)[33], Susan Millard (DeVos Children's Butterworth Hospital, Spectrum Health Systems)[27], Peter Mogayzel (Johns Hopkins University)[27], Richard Moss (Stanford University)[27], Tanda Murray (Johns Hopkins University)[30], Dennis Nielson (University of California at San Francisco)[27], Blakeslee Noyes (Cardinal Glennon Children's Hospital)[27], Wanda O'Neal (University of North Carolina Chapel Hill)[26], David Orenstein (Children's Hospital of Pittsburgh)[27], Brian O'Sullivan (University of Massachusetts Memorial Health Care)[27], Rhonda Pace (University of North Carolina Chapel Hill)[26], Peter Pare (St. Paul's Hospital)[30], H. Worth Parker (Dartmouth-Hitchcock Medical Center, New Hampshire Cystic Fibrosis Center)[27], Mary Ann Passero (Rhode Island Hospital)[27], Elizabeth Perkett (Vanderbilt University)[27], Adrienne Prestridge (Children's Memorial Hospital)[27], Nicholas M. Rafaels (Johns Hopkins University)[30], Bonnie Ramsey (Seattle Children's Hospital, University of Washington)[27], Elizabeth Regan (National Jewish Health)[24], Clement Ren (University of Rochester)[27], George Retsch-Bogart (University of North Carolina Chapel Hill)[27], Michael Rock (University of Wisconsin Hospital and Clinics)[27], Antony Rosen (Johns Hopkins University)[32], Margaret Rosenfeld (Seattle Children's Hospital, University of Washington)[27], Ingo Ruczinski (Johns Hopkins University School of Public Health)[13, 15, 30], Andrew Sanford (University of British Columbia)[30], David Schaeffer (Nemours Children's Clinic)[27], Cindy Sell (University of North Carolina Chapel Hill)[26], Daniel Sheehan (Children's Hospital of Buffalo)[27], Edwin K. Silverman (Brigham and Women's Hospital, Harvard Medical School)[24, 30], Don Sin (Children's Medical Center of Dayton)[30], Terry Spencer (Elliot Health System)[27], Jackie Stonebraker (University of North Carolina Chapel Hill)[26], Holly K. Tabor (Seattle Children's Hospital, University of Washington)[6, 10, 11, 17, 27], Laurie Varlotta (St. Christopher's Hospital for Children)[27], Candelaria I. Vergara (Johns Hopkins University)[30], Robert Weiss[30], Fred Wigley (Johns Hopkins University)[32], Robert A. Wise (Johns Hopkins University)[30], Fred A. Wright (University of North Carolina Chapel Hill)[26], Mark M. Wurfel (University of Washington)[10, 14, 21], Robert Zanni (Monmouth Medical Center)[27], Fei Zou (University of North Carolina Chapel Hill)[26].

Seattle GO:

Deborah A. Nickerson (University of Washington)[3, 4, 5, 7, 8, 9, 11, 15, 17, 18, 19], Mark J. Rieder (University of Washington)[4, 11, 13, 15, 16, 17, 19], Phil Green (University of Washington), Jay Shendure (University of Washington)[1, 8, 14, 16, 17], Joshua M. Akey (University of Washington)[13, 14, 15], Michael J. Bamshad (Seattle Children's Hospital, University of Washington)[4, 6, 7, 8, 10, 11, 13, 15, 17, 27], Carlos D. Bustamante (Stanford University School of Medicine)[3, 13, 15], David R. Crosslin (University of Washington)[2, 9], Evan E. Eichler (University of Washington)[19], P. Keolu Fox[2], Wenqing Fu (University of Washington)[13], Adam Gordon (University of Washington)[11], Simon Gravel (Stanford University School of

Medicine)[13, 15], Gail P. Jarvik (University of Washington)[9, 15], Jill M. Johnsen (Puget Sound Blood Center, University of Washington)[2], Mengyuan Kan (Baylor College of Medicine)[13], Eimear E. Kenny (Stanford University School of Medicine)[3, 13, 15], Jeffrey M. Kidd (Stanford University School of Medicine)[13, 15], Fremiet Lara-Garduno (Baylor College of Medicine)[15], Suzanne M. Leal (Baylor College of Medicine)[1, 13, 15, 16, 17, 19, 20], Dajiang J. Liu (Baylor College of Medicine)[13, 15], Sean McGee (University of Washington)[13, 15, 19], Timothy D. O'Connor (University of Washington)[13], Bryan Paeper (University of Washington)[16], Peggy D. Robertson (University of Washington)4, Joshua D. Smith (University of Washington)[4, 16, 19], Jeffrey C. Staples (University of Washington), Jacob A. Tennessen (University of Washington)[13], Emily H. Turner (University of Washington)[4, 16], Gao Wang (Baylor College of Medicine)[1,13,20], Qian Yi (University of Washington)[4].

WHISP:

Rebecca Jackson (Ohio State University)[1, 2, 4, 5, 8, 12, 14, 15, 17, 18, 20, 34], Kari North (University of North Carolina Chapel Hill)[1, 3, 9, 10, 13, 15, 17, 34], Ulrike Peters (Fred Hutchinson Cancer Research Center)[1, 3, 11, 12, 13, 15, 17, 18, 34], Christopher S. Carlson (Fred Hutchinson Cancer Research Center, University of Washington)[1, 2, 3, 5, 12, 13, 14, 15, 16, 17, 18, 19, 34], Garnet Anderson (Fred Hutchinson Cancer Research Center)[34], Hoda Anton-Culver (University of California at Irvine)[34], Themistocles L. Assimes (Stanford University School of Medicine)[5, 9, 11, 34], Paul L. Auer (Fred Hutchinson Cancer Research Center)[1, 2, 3, 5, 11, 12, 13, 15, 16, 18, 34], Shirley Beresford (Fred Hutchinson Cancer Research Center)[34], Chris Bizon (University of North Carolina Chapel Hill)[3, 9, 13, 15, 34], Henry Black (Rush Medical Center)[34], Robert Brunner (University of Nevada)[34], Robert Brzyski (University of Texas Health Science Center San Antonio)[34], Dale Burwen (National Heart, Lung, and Blood Institute WHI Project Office)[34], Bette Caan (Kaiser Permanente Division of Research, Oakland, CA)[34], Cara L. Carty (Fred Hutchinson Cancer Research Center)[18, 34], Rowan Chlebowski (Los Angeles Biomedical Research Institute)[34], Steven Cummings (University of California at San Francisco)[34], J. David Curb* (University of Hawaii)[9, 18, 34], Charles B. Eaton (Brown University, Memorial Hospital of Rhode Island)[12, 34], Leslie Ford (National Heart, Lung, and Blood Institute, National Heart, Lung, and Blood Institute WHI Project Office)[34], Nora Franceschini (University of North Carolina Chapel Hill)[2, 3, 9, 10, 15, 34], Stephanie M. Fullerton (University of Washington)[6, 11, 34], Margery Gass (University of Cincinnati)[34], Nancy Geller (National Heart, Lung, and Blood Institute WHI Project Office)[34], Gerardo Heiss (University of North Carolina Chapel Hill)[5, 34], Barbara V. Howard (Howard University, MedStar Research Institute)[34], Li Hsu (Fred Hutchinson Cancer Research Center)[1, 13, 15, 18, 34], Carolyn M. Hutter (Fred Hutchinson Cancer Research Center)[13, 15, 18, 34], John Ioannidis (Stanford University School of Medicine)[11, 34], Shuo Jiao (Fred Hutchinson Cancer Research Center)[34], Karen C. Johnson (University of Tennessee Health Science Center)[3, 34], Charles Kooperberg (Fred Hutchinson Cancer Research Center)[1, 5, 9, 13, 14, 15, 17, 18, 34], Lewis Kuller (University of Pittsburgh)[34], Andrea LaCroix (Fred Hutchinson Cancer Research Center)[34], Kamakshi Lakshminarayan (University of Minnesota)[18, 34], Dorothy Lane (State University of New York at Stony Brook)[34], Ethan M. Lange (University of North Carolina Chapel Hill)[3, 5, 9, 13, 34], Leslie A. Lange (University of North Carolina Chapel Hill)[1, 2, 3, 5, 9, 12, 13, 15, 17, 18, 20, 25, 34], Norman Lasser (University of Medicine and Dentistry of New Jersey)[34], Erin LeBlanc

(Kaiser Permanente Center for Health Research, Portland, OR)[34], Cora E. Lewis (University of Alabama at Birmingham)[25,34], Kuo-Ping Li (University of North Carolina Chapel Hill)[9, 34], Marian Limacher (University of Florida)[34], Dan-Yu Lin (University of North Carolina Chapel Hill)[1, 3, 9, 13, 15, 34], Benjamin A. Logsdon (Fred Hutchinson Cancer Research Center)[2, 34], Shari Ludlam (National Heart, Lung, and Blood Institute WHI Project Office)[34], JoAnn E. Manson (Brigham and Women's Hospital, Harvard School of Public Health)[34], Karen Margolis (University of Minnesota)[34], Lisa Martin (George Washington University Medical Center)[9, 34], Joan McGowan (National Heart, Lung, and Blood Institute WHI Project Office)[34], Keri L. Monda (Amgen, Inc.)[1, 15, 34], Jane Morley Kotchen (Medical College of Wisconsin)[34], Lauren Nathan (University of California Los Angeles)[34], Judith Ockene (Fallon Clinic, University of Massachusetts)[34], Mary Jo O'Sullivan (University of Miami)[34], Lawrence S. Phillips (Emory University)[34], Ross L. Prentice (Fred Hutchinson Cancer Research Center)[34], Alexander P. Reiner (Fred Hutchinson Cancer Research Center, University of Washington)[1, 2, 3, 5, 9, 11, 12, 13, 14, 15, 20, 25, 34], John Robbins (University of California at Davis)[34], Jennifer G. Robinson (University of Iowa)[9, 11, 18, 34], Jacques E. Rossouw (National Heart, Lung, and Blood Institute, National Heart, Lung, and Blood Institute WHI Project Office)[5, 14, 17, 20, 34], Haleh Sangi-Haghpeykar (Baylor College of Medicine)[34], Gloria E. Sarto (University of Wisconsin)[34], Sally Shumaker (Wake Forest University)[34], Michael S. Simon (Wayne State University)[34], Marcia L. Stefanick (Stanford University School of Medicine)[34], Evan Stein (Medical Research Labs)[34], Hua Tang (Stanford University)[2, 34], Kira C. Taylor (University of Louisville)[1, 3, 13, 15, 20, 34], Cynthia A. Thomson (University of Arizona)[34], Timothy A. Thornton (University of Washington)[13, 15, 18, 34], Linda Van Horn (Northwestern University)[34], Mara Vitolins (Wake Forest University)[34], Jean Wactawski-Wende (University of Buffalo)[34], Robert Wallace (University of Iowa)[2, 34], Sylvia Wassertheil-Smoller (Boston University)[18, 34], Donglin Zeng (University of North Carolina Chapel Hill)[9, 34].

NHLBI GO ESP Project Team:

Deborah Applebaum-Bowden (National Heart, Lung, and Blood Institute)[4, 7, 12, 17], Michael Feolo (National Center for Biotechnology Information)[12], Weiniu Gan (National Heart, Lung, and Blood Institute)[7, 8, 16, 17], Dina N. Paltoo (National Heart, Lung, and Blood Institute)[4, 6, 11, 17], Jacques E. Rossouw (National Heart, Lung, and Blood Institute, National Heart, Lung, and Blood Institute WHI Project Office)5[, 14, 17, 20, 34], Phyliss Sholinsky (National Heart, Lung, and Blood Institute)[4, 12, 17], Anne Sturcke (National Center for Biotechnology Information)[12].

*deceased

ESP Groups:

[1]Anthropometry Project Team, [2]Blood Count/Hematology Project Team, [3]Blood Pressure Project Team, [4]Data Flow Working Group, [5]Early MI Project Team, [6]ELSI Working Group, [7]Executive Committee, [8]Family Study Project Team, [9]Lipids Project Team, [10]Lung Project Team, [11]Personal Genomics Project Team, [12]Phenotype and Harmonization Working Group, [13]Population Genetics and Statistical Analysis Working Group, [14]Publications and

Presentations Working Group, [15]Quantitative Analysis Ad Hoc Task Group, [16]Sequencing and Genotyping Working Group, [17]Steering Committee, [18]Stroke Project Team, [19]Structural Variation Working Group, [20]Subclinical/Quantitative Project Team

ESP Cohorts:
[21]Acute Lung Injury (ALI), [22]Atherosclerosis Risk in Communities (ARIC), [23]Cardiovascular Health Study (CHS), [24]Chronic Obstructive Pulmonary Disease (COPDGene), [25]Coronary Artery Risk Development in Young Adults (CARDIA), [26]Cystic Fibrosis (CF), [27]Early Pseudomonas Infection Control (EPIC), [28]Framingham Heart Study (FHS), [29]Jackson Heart Study (JHS), [30]Lung Health Study (LHS), [31]Multi-Ethnic Study of Atherosclerosis (MESA), [32]Pulmonary Arterial Hypertension (PAH), [33]Severe Asthma Research Program (SARP), [34]Women's Health Initiative (WHI).

Supplementary Figure 1. Putative damaging variants with MAF < 5% in *SLC22A12*

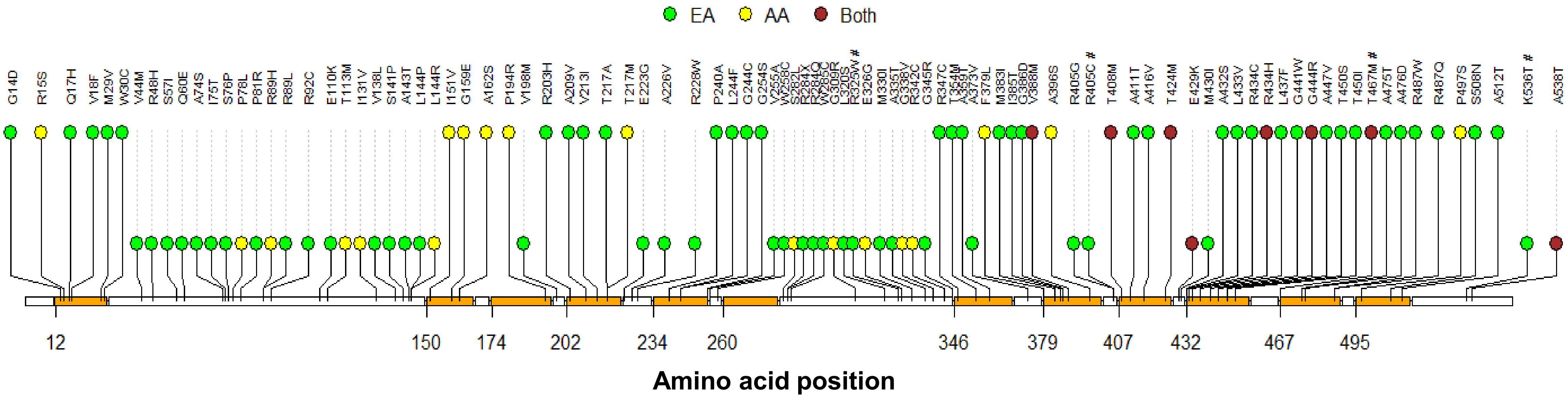

This plot includes 96 of the 97 variants in the primary gene-based test with MAF < 5%.
All 97 variants had MAF < 1%. In *SLC22A12*, no putative damaging variants had MAF between 1 to 5%.
The variant 11:64361276:G:A (MAC=1) is a splice variant and thus not included in this plot.
Orange: predicted transmembrane domains
# denotes the variants selected for functional experiments
Amino acid position based on NP_653186 (ENSP00000366797)

Supplementary Figure 2. Workflow for selecting rare variants in *SLC22A12* for functional study

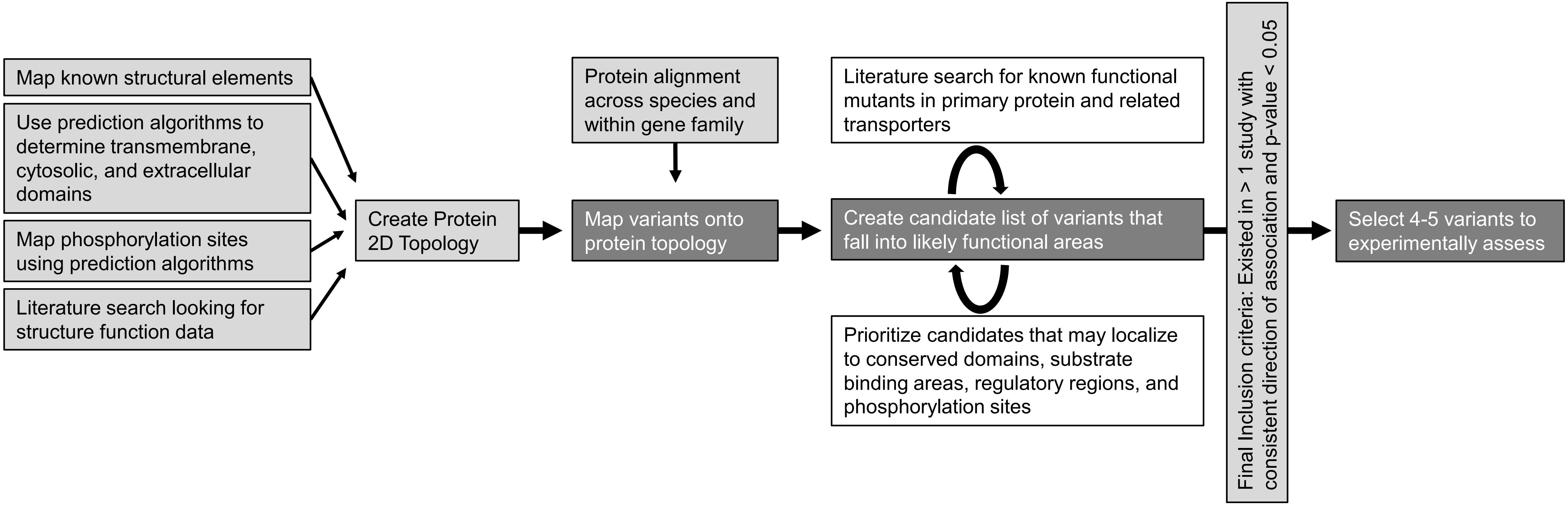

Supplementary Figure 3

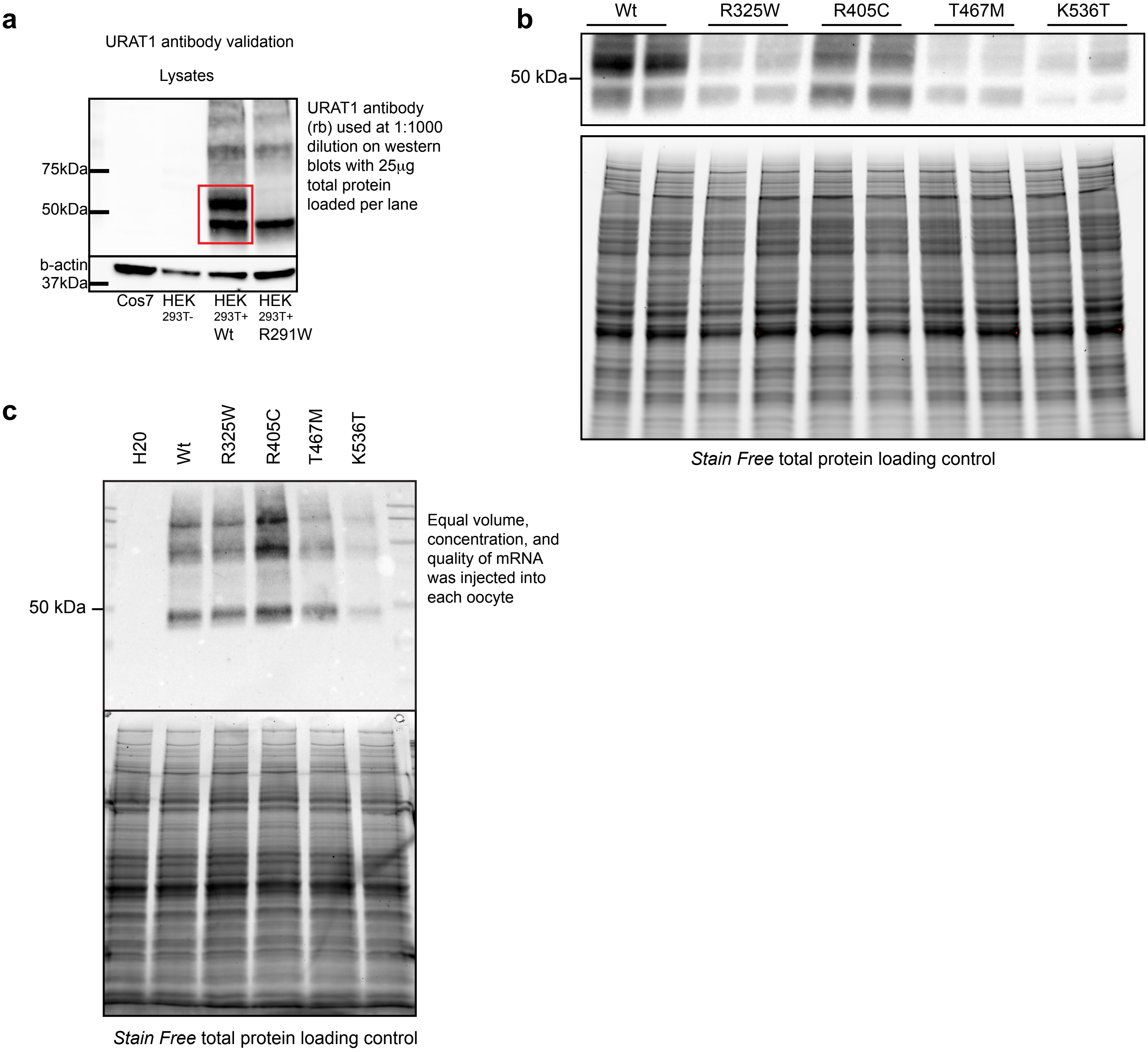

Supplementary Figure 3: Supplemental URAT1 antibody and western blot data
a: URAT1 antibody validation on transiently expressed URAT1 constructs in HEK293T cells and untransfected lysate from COS7 cells. Typical monomer quantification area shown as red box.
b: Total lane protein for Western blot shown in Figure 2B, used as the loading control for quantification (see methods).
c: Full blot and total lane protein for Western blot of URAT1 variants expressed in *Xenopus* oocytes shown in Figure 2C, used as the loading control for quantification (see methods).

Supplementary Figure 4

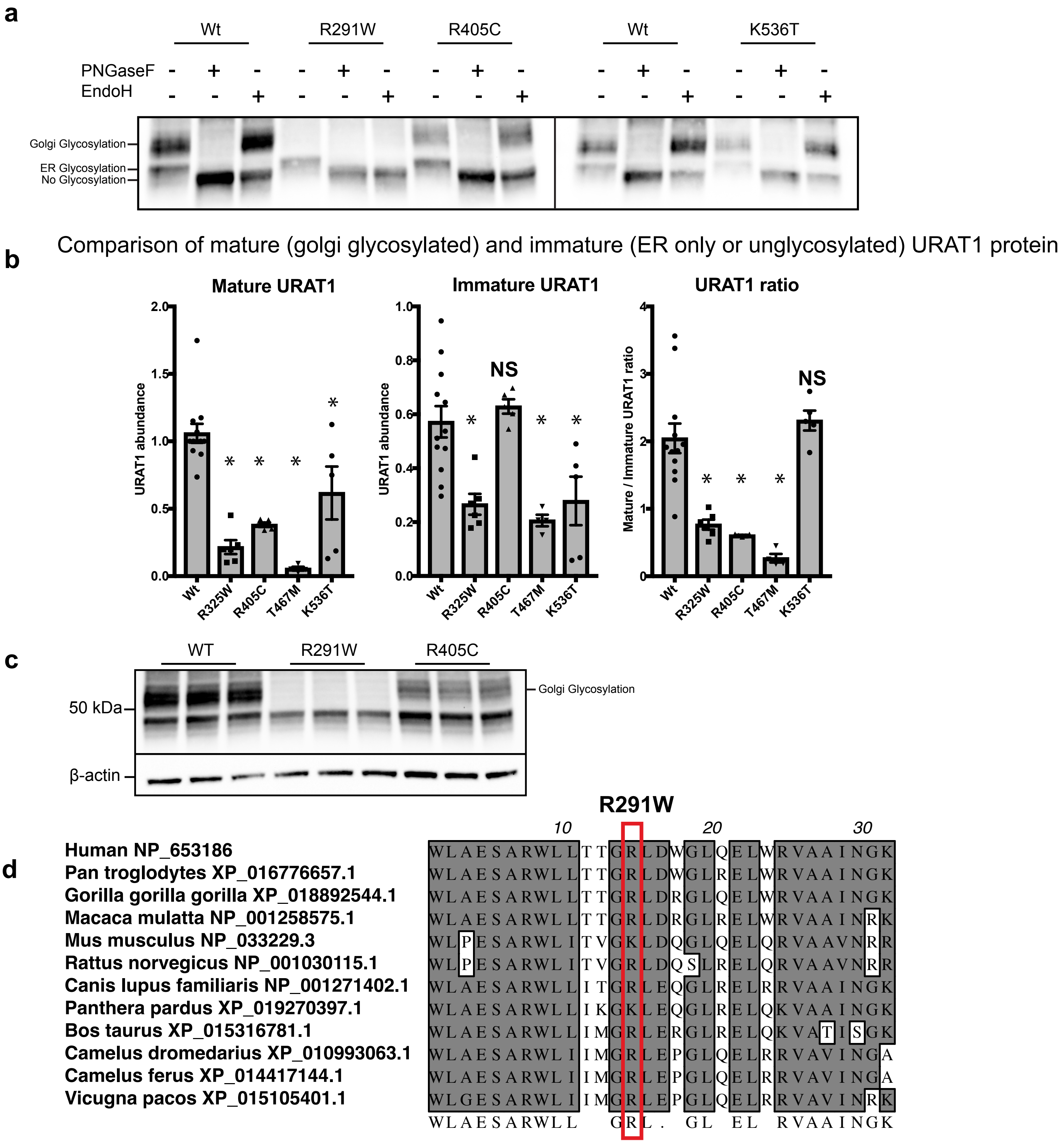

Supplementary Figure 4: The glycosylation and trafficking of URAT1

a: Glycosylation of URAT1 and URAT1 mutants revealed by the PNGase and EndoH enzymes on URAT1 protein transiently expressed in HEK293T cell, representative of n=3.

b: Analysis of the mature (golgi glycosylated) URAT1, immature (ER only or unglycosylated) URAT1, and the mature to immature ratio (+/- SEM, n=5-12, P<0.01). Analysis reveals probable processing / trafficking error for the R325W, R405C, T467M mutants. All Western blots processed in parallel.

c: The R291W mutation results in no protein with complex Golgi associated glycosylation when URAT1 is transiently expressed in HEK293T cells; n=3.

d: Alignment of represenative vertebrate URAT1 proteins demonstrating the conserved nature of the R291 residue.

Supplementary Figure 5

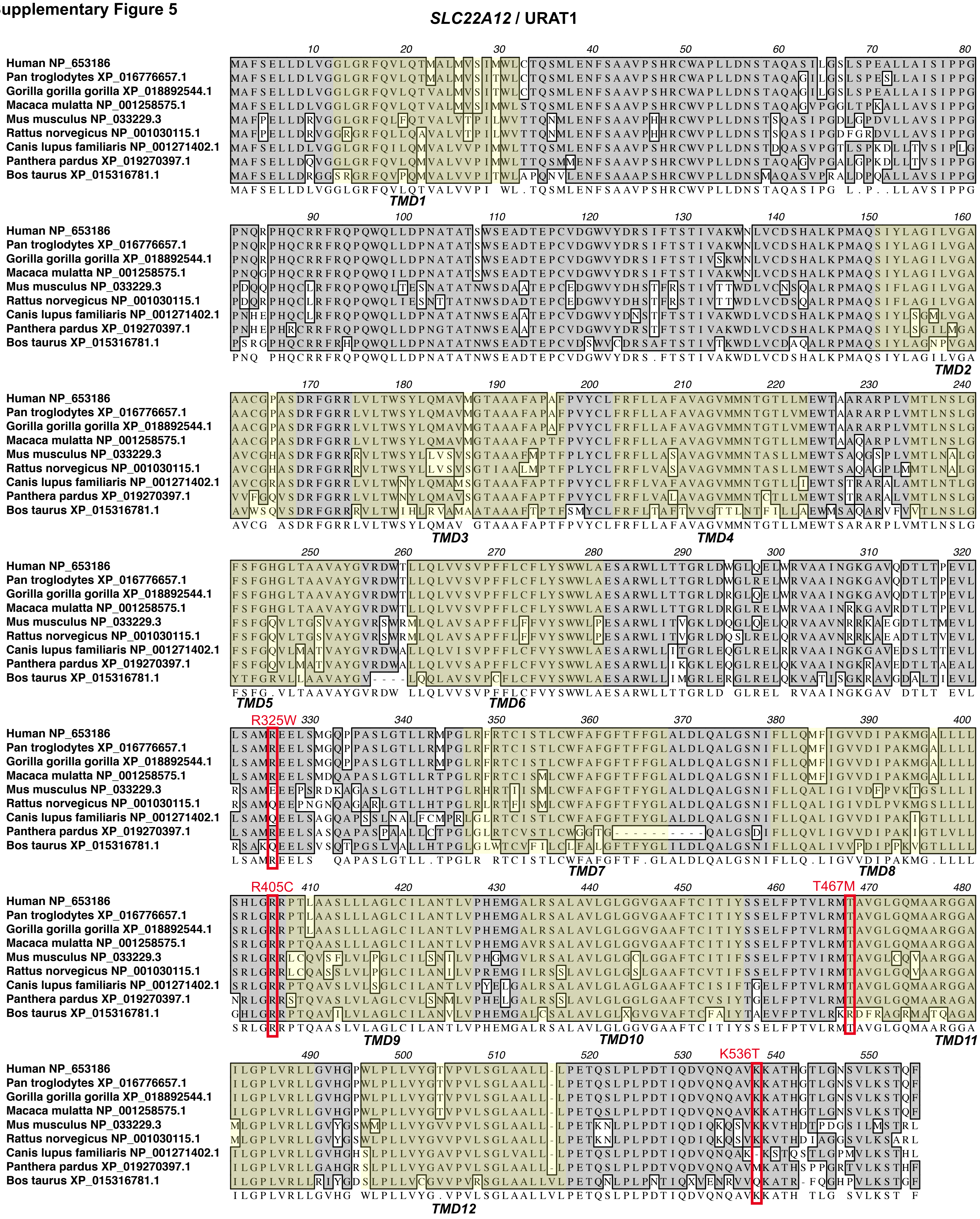

Supplementary Figure 5: Alignment of *SLC22A12* / URAT1
Included are human URAT1 and representative URAT1 sequences of other mammals and the amino acid position of significant variants.

Supplementary Figure 6

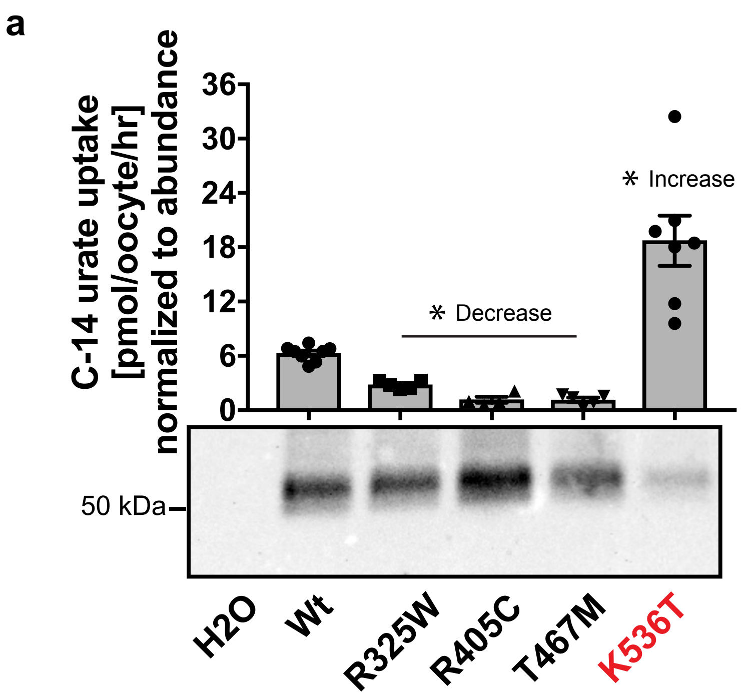

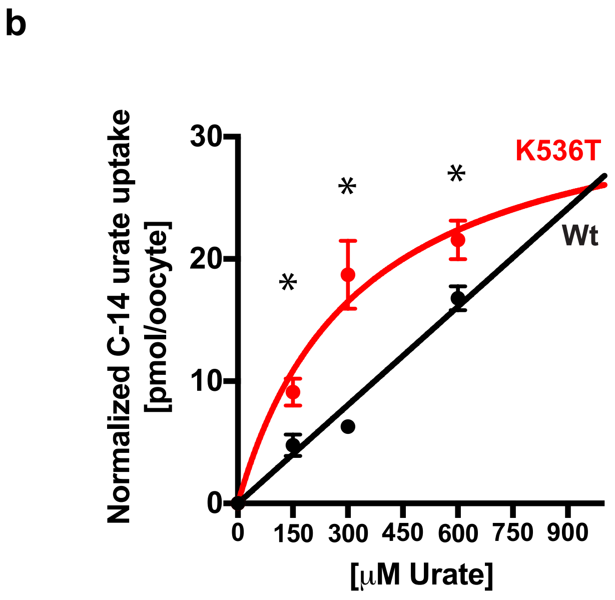

Supplementary Figure 6: Urate transport normalized

a: URAT1 mediated urate transport rates **normalized** to URAT1 protein expression (see Western blot below; lysates from 4-5 pooled oocytes) with the H_2O injected control transport rate subtracted; n=10; +/- SEM; * p<0.01.

b: The concentration dependence on the rate of transport; n=6 for each concentration; +/- SEM; *p<0.05.

Supplementary Figure 7

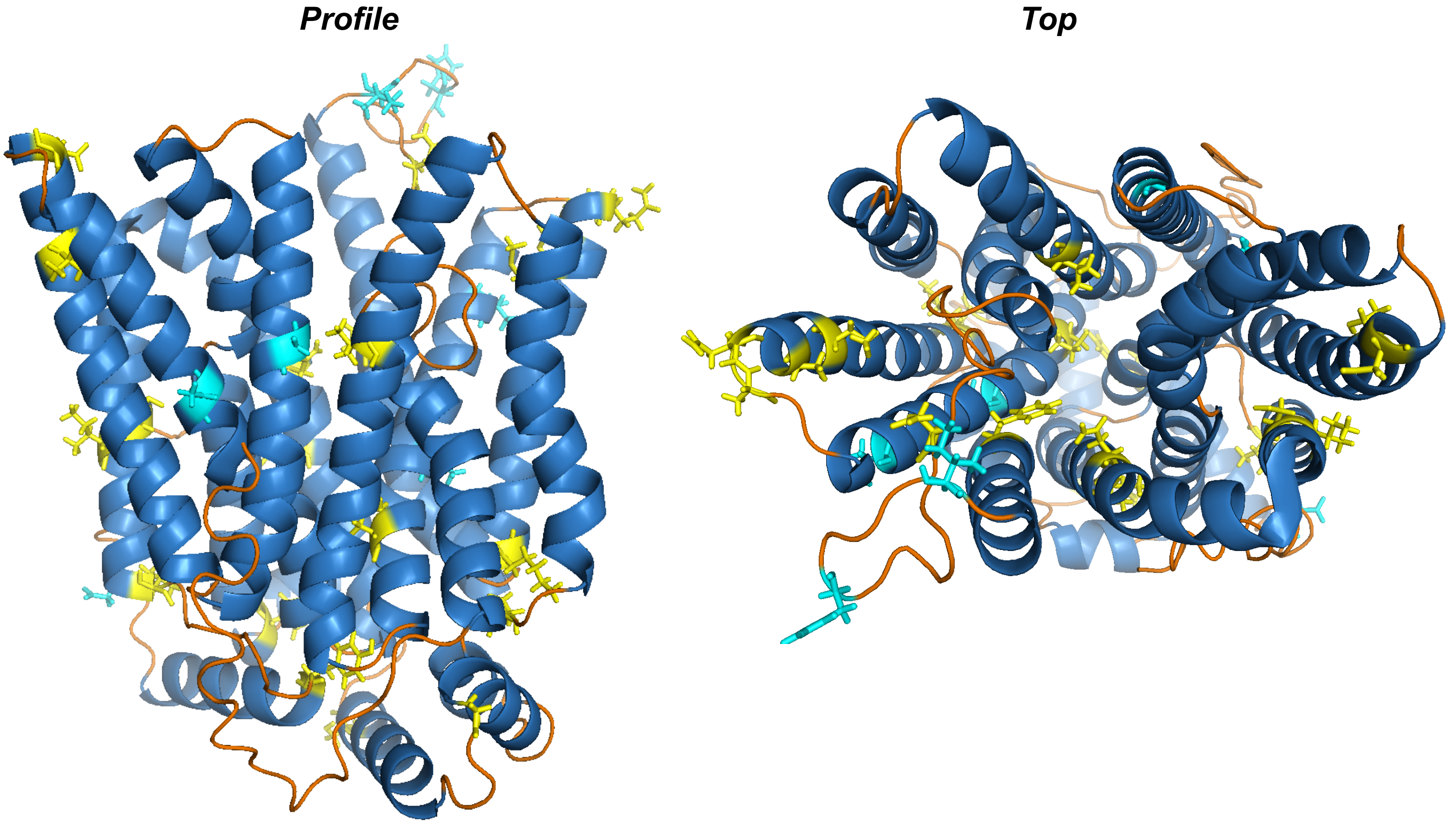

Supplementary Figure 7: Model of SLC2A9b with newly identified variants
Human SLC2A9b model in the outside open configuration, based on GLUT5 crystal (see methods). All residues depicted are Wt residues. Residues depicted with side chains (sticks) are residues with newly identified SLC2A9 variants with absolute effect >1 standard deviations. Yellow sticks = position of variants associated with lower SUA (Wt residue shown); Cyan = position of variants associated with higher SUA (Wt residue shown).

Supplementary Figure 8

SLC2A9 / GLUT9

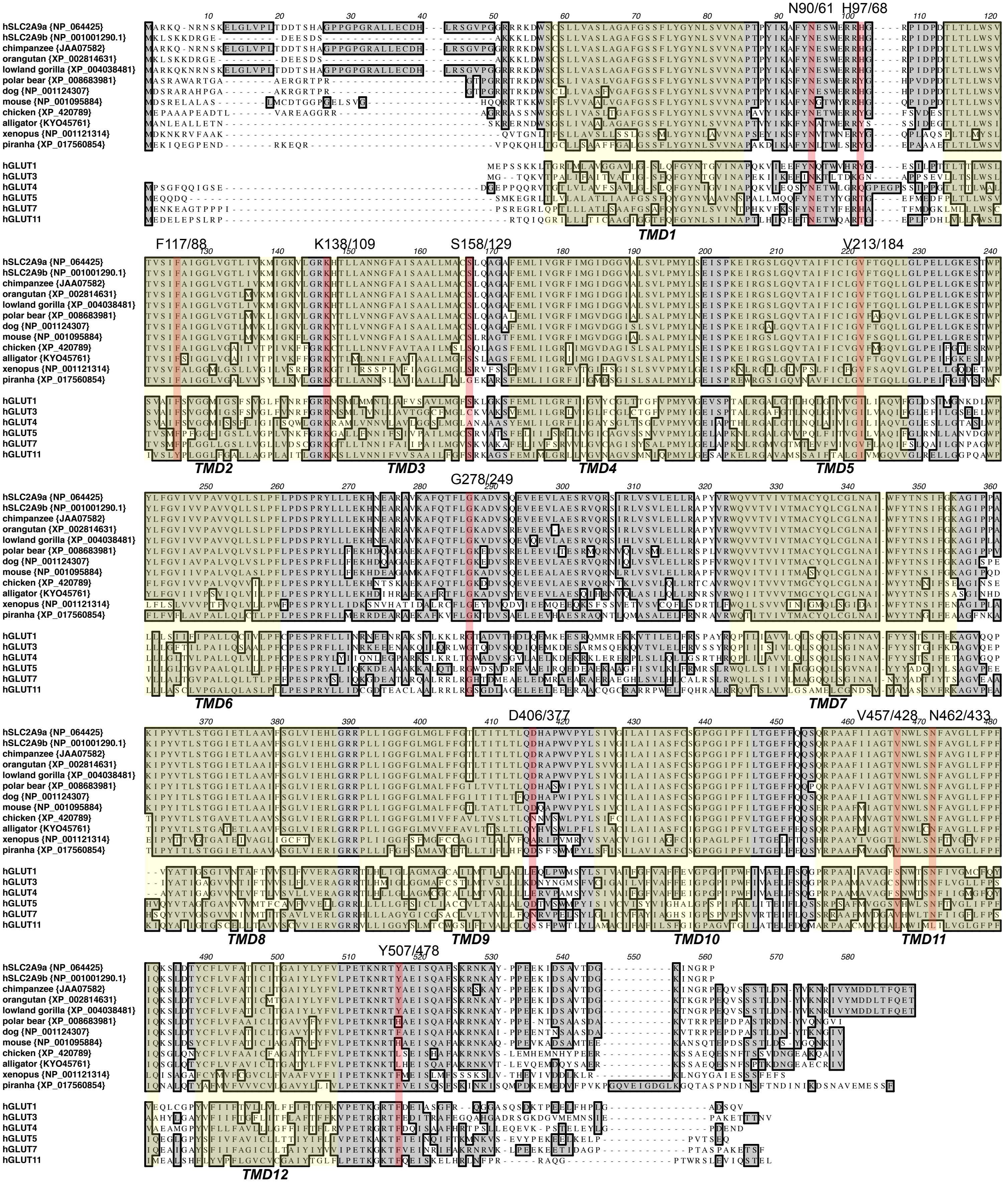

Supplementary Figure 8a: Alignment of *SLC2A9* / GLUT9

Both human isoforms are included and the amino acid position of significant variants labeled in the numbering scheme of SLC2A9a /SLC2A9b. Included are representative GLUT9 sequences of other mammals and other human GLUT family members [1,3,4,5,7, and 11].

Supplementary Figure 8b. Putative damaging variants with MAF < 5% in *SLC2A9* (hSLC2A9a)

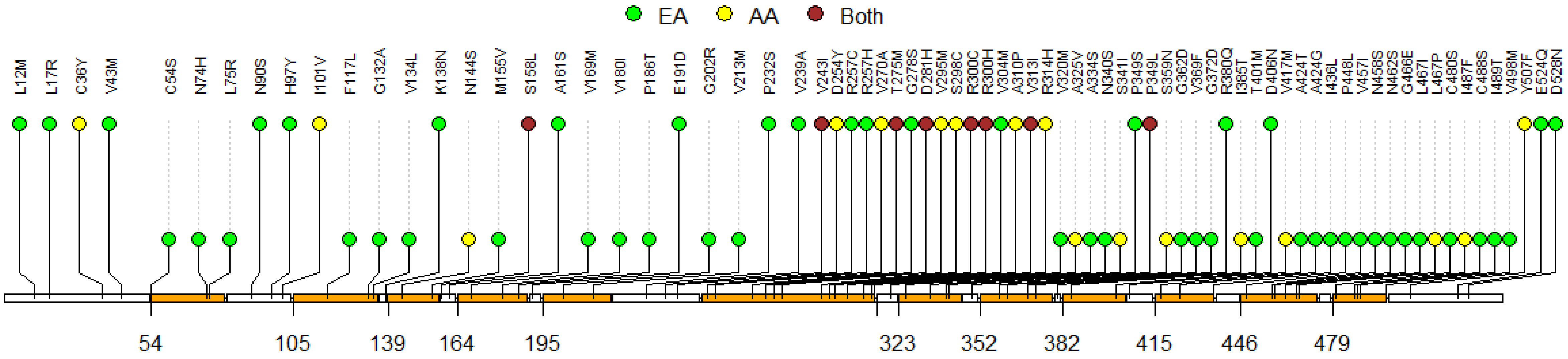

Of the 90 variants included in primary gene-based test, 87 were missense variants, and 3 were splice variants.

Of the missense variants, 76 were found in the hSLC2A9a.

Orange: predicted transmembrane domains

Amino acid position based on NP_064425 (ENSP00000264784)

Supplementary Figure 9

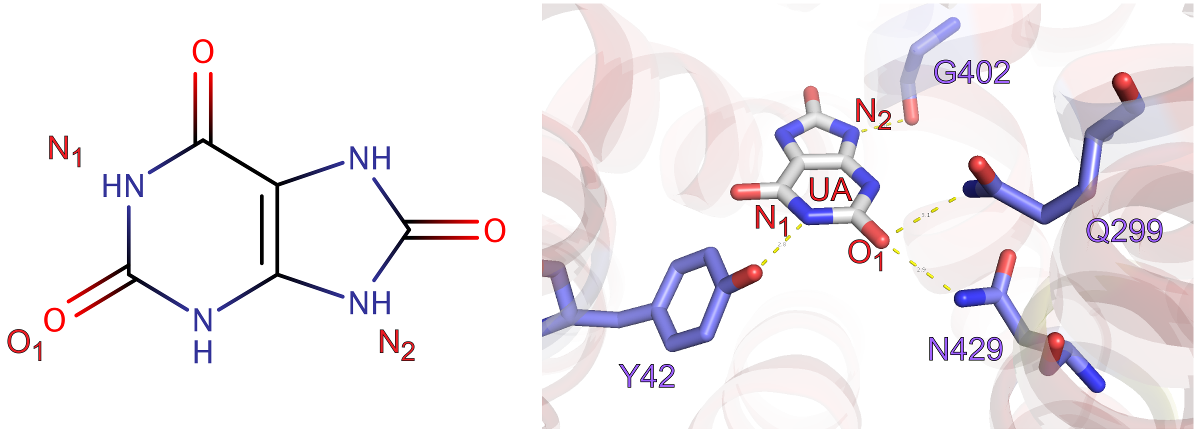

Supplementary Figure 9: Uric acid and the modeled binding pocket of human SLC2A9b
Uric acid molecule schematic and structural model with the key Nitrogens and Oxygen atoms labeled corresponding to nomenclature of the uric acid binding pocket as depicted in Figure 3D.

Supplementary Figure 10. Flow chart of meta-analyses for serum urate, eGFR, and UACR

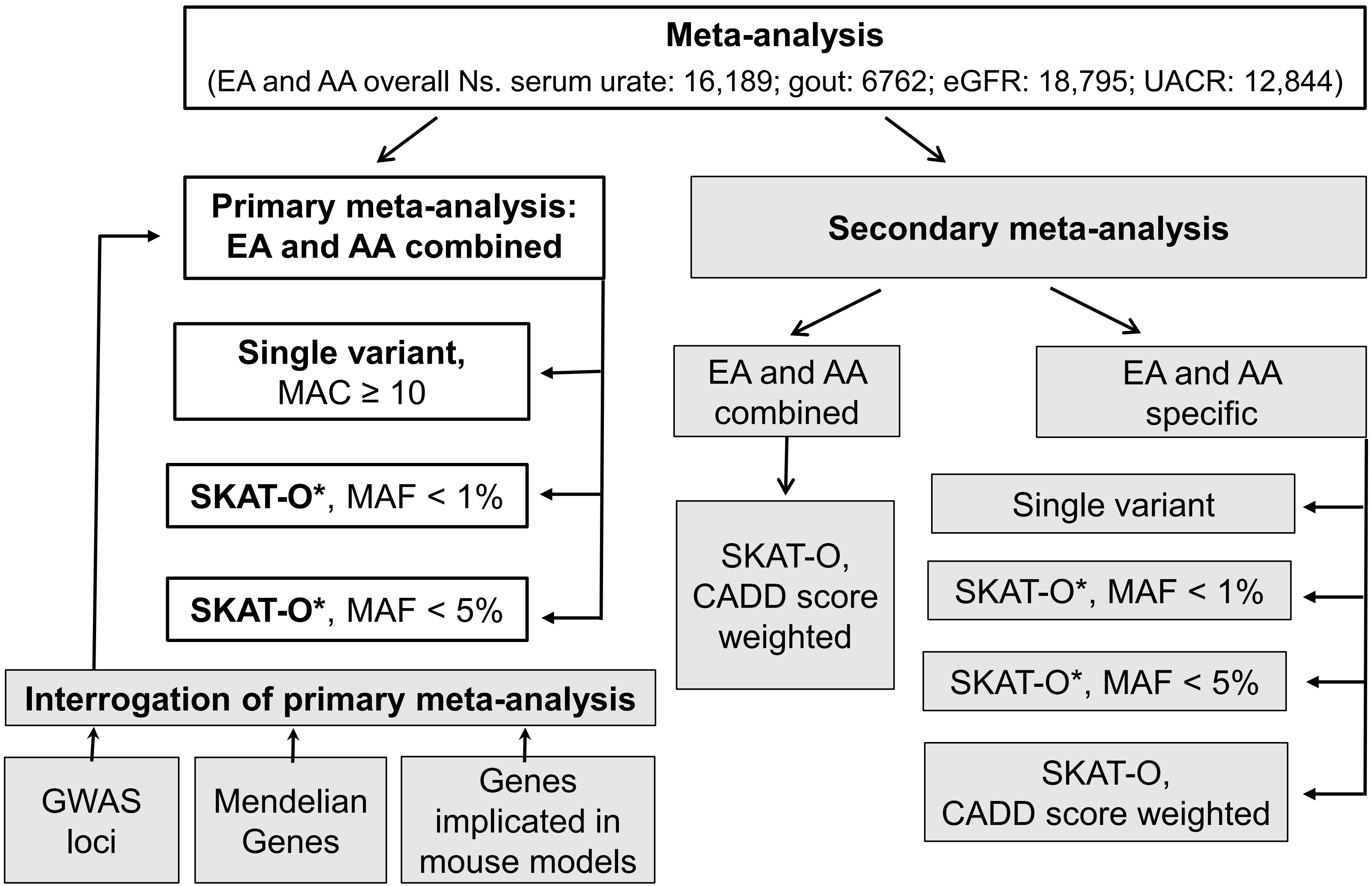

SKAT-O*: Optimal Sequence Kernel Association Test weight by beta distribution (1, 25). In addition to the MAF filter, only genes with two or more variants were included;
CADD: Combined Annotation–Dependent Depletion.
Total number of variants with MAC ≥ 10 in primary single variant meta-analysis: 781,062 in 19,549 genes.

References

1. Kircher, M., Witten, D.M., Jain, P., O'Roak, B.J., Cooper, G.M., and Shendure, J. (2014). A general framework for estimating the relative pathogenicity of human genetic variants. Nat Genet 46, 310-315.
2. Feig, D.I., Kang, D.H., Nakagawa, T., Mazzali, M., and Johnson, R.J. (2006). Uric acid and hypertension. Curr Hypertens Rep 8, 111-115.
3. Sundstrom, J., Sullivan, L., D'Agostino, R.B., Levy, D., Kannel, W.B., and Vasan, R.S. (2005). Relations of serum uric acid to longitudinal blood pressure tracking and hypertension incidence. Hypertension 45, 28-33.
4. Sedaghat, S., Pazoki, R., Uitterlinden, A.G., Hofman, A., Stricker, B.H., Ikram, M.A., Franco, O.H., and Dehghan, A. (2014). Association of uric acid genetic risk score with blood pressure: the Rotterdam study. Hypertension 64, 1061-1066.
5. Yu, B., Pulit, S.L., Hwang, S.J., Brody, J.A., Amin, N., Auer, P.L., Bis, J.C., Boerwinkle, E., Burke, G.L., Chakravarti, A., et al. (2016). Rare Exome Sequence Variants in CLCN6 Reduce Blood Pressure Levels and Hypertension Risk. Circ Cardiovasc Genet 9, 64-70.
6. Firmann, M., Mayor, V., Vidal, P.M., Bochud, M., Pecoud, A., Hayoz, D., Paccaud, F., Preisig, M., Song, K.S., Yuan, X., et al. (2008). The CoLaus study: a population-based study to investigate the epidemiology and genetic determinants of cardiovascular risk factors and metabolic syndrome. BMC Cardiovasc Disord 8, 6.
7. Jurgens, C., Volzke, H., and Tost, F. (2014). [Study of health in Pomerania (SHIP-Trend): : Important aspects for healthcare research in ophthalmology]. Ophthalmologe 111, 443-447.
8. Enomoto, A., Kimura, H., Chairoungdua, A., Shigeta, Y., Jutabha, P., Cha, S.H., Hosoyamada, M., Takeda, M., Sekine, T., Igarashi, T., et al. (2002). Molecular identification of a renal urate anion exchanger that regulates blood urate levels. Nature 417, 447-452.
9. Ichida, K., Hosoyamada, M., Hisatome, I., Enomoto, A., Hikita, M., Endou, H., and Hosoya, T. (2004). Clinical and molecular analysis of patients with renal hypouricemia in Japan-influence of URAT1 gene on urinary urate excretion. J Am Soc Nephrol 15, 164-173.
10. Iwai, N., Mino, Y., Hosoyamada, M., Tago, N., Kokubo, Y., and Endou, H. (2004). A high prevalence of renal hypouricemia caused by inactive SLC22A12 in Japanese. Kidney Int 66, 935-944.
11. Wakida, N., Tuyen, D.G., Adachi, M., Miyoshi, T., Nonoguchi, H., Oka, T., Ueda, O., Tazawa, M., Kurihara, S., Yoneta, Y., et al. (2005). Mutations in human urate transporter 1 gene in presecretory reabsorption defect type of familial renal hypouricemia. J Clin Endocrinol Metab 90, 2169-2174.
12. Vazquez-Mellado, J., Alvarado-Romano, V., Burgos-Vargas, R., Jimenez-Vaca, A.L., Pozo-Molina, G., and Cuevas-Covarrubias, S.A. (2007). Homozygous frameshift mutation in the SLC22A12 gene in a patient with primary gout and high levels of serum uric acid. J Clin Pathol 60, 947-948.
13. Lam, C.W., Kong, A.P., Tsui, T.K., Ozaki, R., Chan, H.M., Tong, S.F., Siu, T.S., Tam, S., and Chan, J.C. (2008). A novel mutation of SLC22A12 gene causing primary renal hypouricemia in a patient with metabolic syndrome. Clin Chim Acta 398, 157-158.
14. Matsuo, H., Chiba, T., Nagamori, S., Nakayama, A., Domoto, H., Phetdee, K., Wiriyasermkul, P., Kikuchi, Y., Oda, T., Nishiyama, J., et al. (2008). Mutations in

glucose transporter 9 gene SLC2A9 cause renal hypouricemia. Am J Hum Genet 83, 744-751.
15. Dinour, D., Gray, N.K., Campbell, S., Shu, X., Sawyer, L., Richardson, W., Rechavi, G., Amariglio, N., Ganon, L., Sela, B.A., et al. (2010). Homozygous SLC2A9 mutations cause severe renal hypouricemia. J Am Soc Nephrol 21, 64-72.
16. Dinour, D., Bahn, A., Ganon, L., Ron, R., Geifman-Holtzman, O., Knecht, A., Gafter, U., Rachamimov, R., Sela, B.A., Burckhardt, G., et al. (2011). URAT1 mutations cause renal hypouricemia type 1 in Iraqi Jews. Nephrol Dial Transplant 26, 2175-2181.
17. Tasic, V., Hynes, A.M., Kitamura, K., Cheong, H.I., Lozanovski, V.J., Gucev, Z., Jutabha, P., Anzai, N., and Sayer, J.A. (2011). Clinical and functional characterization of URAT1 variants. PLoS One 6, e28641.
18. Dinour, D., Gray, N.K., Ganon, L., Knox, A.J., Shalev, H., Sela, B.A., Campbell, S., Sawyer, L., Shu, X., Valsamidou, E., et al. (2012). Two novel homozygous SLC2A9 mutations cause renal hypouricemia type 2. Nephrol Dial Transplant 27, 1035-1041.
19. Li, Z., Ding, H., Chen, C., Chen, Y., Wang, D.W., and Lv, Y. (2013). Novel URAT1 mutations caused acute renal failure after exercise in two Chinese families with renal hypouricemia. Gene 512, 97-101.
20. Stiburkova, B., Sebesta, I., Ichida, K., Nakamura, M., Hulkova, H., Krylov, V., Kryspinova, L., and Jahnova, H. (2013). Novel allelic variants and evidence for a prevalent mutation in URAT1 causing renal hypouricemia: biochemical, genetics and functional analysis. Eur J Hum Genet 21, 1067-1073.
21. Jeannin, G., Chiarelli, N., Gaggiotti, M., Ritelli, M., Maiorca, P., Quinzani, S., Verzeletti, F., Possenti, S., Colombi, M., and Cancarini, G. (2014). Recurrent exercise-induced acute renal failure in a young Pakistani man with severe renal hypouricemia and SLC2A9 compound heterozygosity. BMC Med Genet 15, 3.
22. Androvitsanea, A., Stylianou, K., Maragkaki, E., Tzanakakis, M., Stratakis, S., Petrakis, I., Giatzakis, C., and Daphnis, E. (2015). Vanishing urate, acute kidney injury episodes and a homozygous SLC2A9 mutation. Int Urol Nephrol 47, 1035-1036.
23. Fujita, K., and Ichida, K. (2016). A novel compound heterozygous mutation in the SLC22A12 (URAT1) gene in a Japanese patient associated with renal hypouricemia. Clin Chim Acta 463, 119-121.
24. Mancikova, A., Krylov, V., Hurba, O., Sebesta, I., Nakamura, M., Ichida, K., and Stiburkova, B. (2016). Functional analysis of novel allelic variants in URAT1 and GLUT9 causing renal hypouricemia type 1 and 2. Clin Exp Nephrol 20, 578-584.
25. Okabayashi, Y., Yamamoto, I., Komatsuzaki, Y., Niikura, T., Yamakawa, T., Katsumata, H., Kawabe, M., Katsuma, A., Nakada, Y., Kobayashi, A., et al. (2016). Rare case of nephrocalcinosis in the distal tubules caused by hereditary renal hypouricaemia 3 months after kidney transplantation. Nephrology (Carlton) 21 Suppl 1, 67-71.
26. Windpessl, M., Ritelli, M., Wallner, M., and Colombi, M. (2016). A Novel Homozygous SLC2A9 Mutation Associated with Renal-Induced Hypouricemia. Am J Nephrol 43, 245-250.
27. Claverie-Martin, F., Trujillo-Suarez, J., Gonzalez-Acosta, H., Aparicio, C., Justa Roldan, M.L., Stiburkova, B., Ichida, K., Martin-Gomez, M.A., Herrero Goni, M., Carrasco Hidalgo-Barquero, M., et al. (2018). URAT1 and GLUT9 mutations in Spanish patients with renal hypouricemia. Clin Chim Acta 481, 83-89.
28. Kawamura, Y., Matsuo, H., Chiba, T., Nagamori, S., Nakayama, A., Inoue, H., Utsumi, Y., Oda, T., Nishiyama, J., Kanai, Y., et al. (2011). Pathogenic GLUT9 mutations

causing renal hypouricemia type 2 (RHUC2). Nucleosides, nucleotides & nucleic acids 30, 1105-1111.
29. NHGRI-EBI. A Catalog of Published Genome-Wide Association Studies (https://www.ebi.ac.uk/gwas/), accessed December 10, 2016.
30. Larsen, C.P., Durfee, T., Wilson, J.D., and Beggs, M.L. (2016). A Custom Targeted Next-Generation Sequencing Gene Panel for the Diagnosis of Genetic Nephropathies. Am J Kidney Dis 67, 992-993.
31. OMIM. Online Mendelian Inheritance in Man (https://www.omim.org/), accessed December 10, 2016.
32. Jing, J., Pattaro, C., Hoppmann, A., Okada, Y., Fox, C.S., and Kottgen, A. (2016). Combination of mouse models and genomewide association studies highlights novel genes associated with human kidney function. Kidney Int 90, 764-773.
33. MGI. Mouse Genome Informatics (MGI) database (http://www.informatics.jax.org), accessed December 10, 2016.
34. HMDC. Human - Mouse: Disease Connection (HMDC), http://www.informatics.jax.org/humanDisease.shtml, accessed December 10, 2016.
35. Xue, Y., Liu, Z., Cao, J., Ma, Q., Gao, X., Wang, Q., Jin, C., Zhou, Y., Wen, L., and Ren, J. (2011). GPS 2.1: enhanced prediction of kinase-specific phosphorylation sites with an algorithm of motif length selection. Protein Eng Des Sel 24, 255-260.
36. Pattaro, C., Teumer, A., Gorski, M., Chu, A.Y., Li, M., Mijatovic, V., Garnaas, M., Tin, A., Sorice, R., Li, Y., et al. (2016). Genetic associations at 53 loci highlight cell types and biological pathways relevant for kidney function. Nat Commun 7, 10023.
37. Li, M., Li, Y., Weeks, O., Mijatovic, V., Teumer, A., Huffman, J.E., Tromp, G., Fuchsberger, C., Gorski, M., Lyytikainen, L.P., et al. (2016). SOS2 and ACP1 Loci Identified through Large-Scale Exome Chip Analysis Regulate Kidney Development and Function. J Am Soc Nephrol.
38. Naik, R.P., Derebail, V.K., Grams, M.E., Franceschini, N., Auer, P.L., Peloso, G.M., Young, B.A., Lettre, G., Peralta, C.A., Katz, R., et al. (2014). Association of sickle cell trait with chronic kidney disease and albuminuria in African Americans. JAMA 312, 2115-2125.
